# Cerebral Embolic Protection Devices (CEPDs) During Transcatheter Aortic Valve Implantation (TAVI): A Meta‐Analysis of Randomized Controlled Trials

**DOI:** 10.1002/clc.70309

**Published:** 2026-04-23

**Authors:** Mohamed Ibrahim Gbreel, Ahmed Samy Badran, Marwa Hassan, Mahmoud Balata

**Affiliations:** ^1^ Faculty of Medicine October 6 University Giza Egypt; ^2^ Faculty of Medicine Ain Shams University Cairo Egypt; ^3^ Theodor Bilharz Research Institute Giza Egypt; ^4^ Department of Cardiology University Hospital Rostock Rostock Germany

**Keywords:** cerebral embolic protection, meta‐analysis, stroke, TAVI, transcatheter aortic valve implantation

## Abstract

**Aims:**

Transcatheter aortic valve implantation (TAVI) is associated with procedure‐related stroke. Cerebral embolic protection devices (CEPDs) are designed to reduce the risk of embolic debris reaching the brain; however, the evidence supporting their efficacy remains controversial. We aim to evaluate the efficacy and safety of CEPDs in patients undergoing TAVI.

**Methods:**

Major databases were systematically searched up to April 2025. Only randomized controlled trials (RCTs) were included and critically appraised using the Cochrane Risk of Bias (ROB‐2) tool. We calculated risk ratios (RRs) with the 95% confidence intervals for the outcomes, and trial sequential analysis (TSA) was conducted to reduce the risk of false‐positive results due to random errors.

**Results:**

Eight RCTs (11,589 patients) were analyzed. No significant difference was observed in overall stroke incidence between CEPD and control groups (0.92 (95% CI: 0.75–1.14; *p* = 0.40, I^2^ = 0%), including disabling and non‐disabling strokes. Device‐specific analyses showed a non‐significant reduction in disabling stroke with the Sentinel device. All‐cause mortality, transient ischemic attacks, bleeding, acute kidney injury, delirium, and pacemaker implantation rates were similar between groups. CEPD use was linked to a transient improvement in cognitive function (MoCA scores) at 2–5 days post‐TAVI, but this effect was not sustained at later follow‐ups. TSA indicated that current evidence is insufficient to definitively refute CEPD efficacy.

**Conclusion:**

CEPDs show no significant reduction in overall, disabling, or non‐disabling stroke, nor in all‐cause mortality post‐TAVI.

**Trial Registration:**

This meta‐analysis was registered on PROSPERO. No.: CRD420251026208.

## Graphical abstract

This meta‐analysis of eight RCTs (n = 11,589) demonstrates that the routine use of cerebral embolic protection devices during TAVI does not significantly reduce the risk of overall, disabling, or non‐disabling stroke, nor does it confer a mortality benefit.

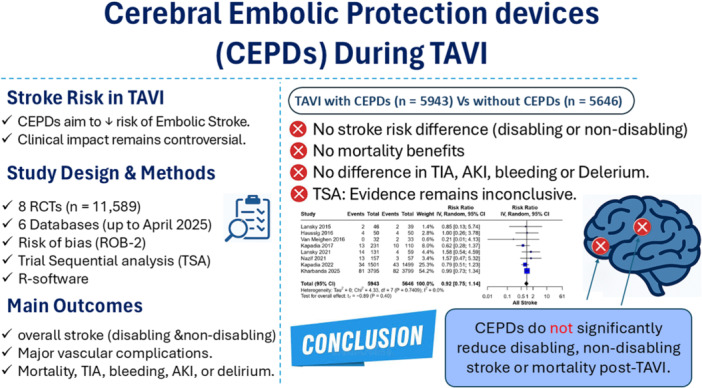

**Abbreviations**
ACC: American College of Cardiology
AF: atrial fibrillation
AKI: acute kidney injury
CAD: coronary artery diseases
CEPD: cerebral embolic protection devices
CES‐D: The Center for Epidemiologic Studies Depression scale
CHF: Congestive heart failure
CI: confidence interval
DWMRI: diffusion‐weighted magnetic resonance imaging
EACTS: European Association for Cardio‐Thoracic Surgery
ESC: European society of cardiology
HTN: hypertension
MACCE: major adverse cardiovascular and cerebrovascular events
MD: mean difference
MMSE: Mini‐Mental State Examination
MoCA: Montreal Cognitive Assessment
NYHA: New York Heart Association
PCI: percutaneous coronary intervention
PRISMA: Preferred Reporting Items for Systematic Reviews and Meta‐Analyses
PVD: Peripheral vascular diseases
RCT: randomized controlled trial
RR: risk ratio
SAVR: surgical aortic valve replacement
STS PROM: Society of Thoracic Surgeons Predicted Risk of Mortality
TAVI: transcatheter aortic valve implantation
TIA: Transient ischemic attack

## 1 Introduction

Transcatheter aortic valve implantation (TAVI) has become a well‐known and established technique for severe aortic stenosis [1, 2], offering a minimally invasive alternative to traditional surgical aortic valve replacement. It provides advantages in terms of mortality, recovery, hospital stay, bleeding, and postoperative pain [2, 3].

Despite the mortality benefit of TAVI, stroke after TAVI has an incidence rate of 3.8% as per the PARTNER trial [4]. Stroke remains an unpredictable, serious complication that compromises survival and functional recovery [5].

Cerebral embolic protection devices (CEPDs) are designed to reduce the risk of embolic stroke by preventing the released debris from reaching the brain [6]. They have been proposed as a potential strategy to lower the risk of post‐TAVI stroke. However, the effectiveness of these devices remains controversial. The first trial to evaluate a CEPD was conducted by Wendt et al.; however, it was prematurely terminated, included a small sample size (30 patients), and reported no incidence of stroke in either the CEPD group or the control group [7]. Over the past decade, several trials have emerged. Most recently, the BHF PROTECT‐TAVI trial by Kharbanda et al. a multicenter study conducted in the UK over 7635 patients, has contributed further evidence to this field [8]. Therefore, we conducted a comprehensive systematic review of randomized controlled trials (RCTs), followed by a meta‐analysis to evaluate the outcomes of using CEPDs during TAVI procedures.

## 2 Materials and Methods

This study followed the Cochrane Handbook of Systematic Reviews on Interventions and the Preferred Reporting Items for Systematic Reviews and Meta‐Analyses (PRISMA) statement guidelines [9, 10] Our protocol was registered on the Prospero registry with an ID (CRD420251026208).

### 2.1 Search Strategy

The following major databases were systematically searched up to April 4, 2025: PubMed, Web of Science (WOS), Scopus, Cochrane Library, Embase, and MEDLINE via the Ovid interface. A comprehensive search strategy was developed using broad terms related to “transcatheter aortic valve replacement” and “cerebral embolic protection,” with detailed search strings for each database provided in Supporting Information S1: Table 1. Additionally, reference lists of the included studies were screened to ensure that no relevant trials were missed. An updated search was performed across all databases on March 10, 2026, identifying no additional eligible RCTs.

### 2.2 Eligibility Criteria

We screened the retrieved records based on predefined PICO criteria, including studies involving patients who underwent TAVI, with the intervention group receiving CEPDs and the control group undergoing TAVI without CEPDs. To ensure the highest quality of evidence, only strictly RCTs were included. Non‐RCTs, including observational studies (cohort and case‐control designs), other study designs, conference abstracts, case reports, case series, and non‐English publications were excluded.

### 2.3 Screening and Study Selection

All retrieved records from the databases were imported into EndNote software [11], where duplicates were identified and removed. The remaining references were screened for relevance in two stages: initial title and abstract screening, followed by full‐text screening to determine final eligibility. All screening steps were conducted independently by two reviewers (M.I.G. and A.S.B.), with any disagreements resolved through discussion or by consulting a third author (M.H).

### 2.4 Data Extraction

Two authors (M.I.G. and A.S.B.) independently reviewed each included study and extracted data using a standardized form, with any disagreements resolved through discussion or by consulting a third author. Extracted information included: authors, year of publication, trial name, study setting, trial registration, inclusion criteria, sample size, type of valve replaced, device used, follow‐up duration, and neurological or neurocognitive assessments. Baseline patient characteristics included demographics, body mass index (BMI), NYHA classification, STS PROM score, and relevant medical history. We also extracted the methodological criteria used to define and adjudicate stroke events in each study.

### 2.5 Quality Assessment and Evidence Rating

For all included RCTs, we used the Cochrane Collaboration's Risk of Bias tool version 2 (RoB 2) [12] to assess methodological quality. This tool evaluates five domains: the randomization process, deviations from intended interventions, missing outcome data, measurement of outcomes, and selection of reported results. Additionally, we assessed the overall quality of evidence from the meta‐analysis using the Grading of Recommendations, Assessment, Development, and Evaluation (GRADE) approach. According to GRADE, five factors may lower the quality of evidence in randomized trials: study limitations, inconsistency, indirectness, imprecision, and publication bias [13, 14]. All quality assessments were performed independently by M.I.G. and A.S.B., with any disagreements resolved through discussion.

### 2.6 Primary and Secondary Endpoints

The primary endpoints were the incidence of stroke and all‐cause mortality. The primary analysis for stroke uesd a pre‐specified periprocedural timepoint (preferentially extracting 30‐day cumulative incidence). When 30‐day data were unavailable, the closest early timepoint was utilized to isolate the procedural risk. To harmonize varying severity definitions, strokes were categorized as disabling or non‐disabling guided by the Valve Academic Research Consortium (VARC) criteria. Secondary endpoints included transient ischemic attack (TIA), life‐threatening or disabling bleeding, major vascular complications (MVCs), and worsening of National Institutes of Health Stroke Scale (NIHSS) and Montreal Cognitive Assessment (MoCA) scores. Additional secondary outcomes included acute kidney injury (AKI), pacemaker implantation, and delirium. Subgroup analyses were conducted to assess outcomes based on variables such as the device used (Sentinel vs. TriGuard), type of stroke, and duration of follow‐up.

### 2.7 Statistical Analysis

We conducted the meta‐analysis in R Studio using R (version 4.4.3) software using the “meta” and “metafor” packages. We pooled the dichotomous data, which were analyzed as a risk ratio (RR) and 95% confidence interval (CI). A significance level of *p* < 0.05, which is also inferred from whether the CI crosses the null. *I* ^2^ values greater than 50% were considered indicators of high heterogeneity. A random‐effects model, using Restricted Maximum Likelihood (REML) as the estimator and the Hartung–Knapp adjustment, was applied for all outcomes. This model was applied due to different CEPD platforms, different valve eras and procedural techniques, variable neuro assessment intensity, and different baseline stroke risks across study populations. For the primary outcome of overall stroke, we used the longest available short‐term follow‐up reported by each trial. Time‐to‐event analysis was precluded by inconsistent hazard ratio reporting across RCTs; thus, pooling relied on cumulative event counts, which appropriately capture procedural risk. Subgroup analyses were conducted by precise follow‐up duration, stroke severity, and device type. We performed Trial Sequential Analysis (TSA) for the primary outcomes, using TSA software (Version 0.9.5.10 Beta, Copenhagen Trial Unit, Copenhagen) [15] to avoid false‐positive results and to draw conclusive evidence [16]. In the TSA, the required information size (RIS) was estimated based on the anticipated effect size of the intervention, assuming a type I error of 5% and a power of 80%. For sensitivity analysis, the power was increased to 90% while maintaining a type I error of 5%. The information axis reflected the cumulative sample size. Threshold adjustments for the *Z*‐score boundaries were made using the O'Brien–Fleming alpha‐spending function. Formal assessment of publication bias and small‐study effects via funnel plots or statistical tests (e.g., Egger's test) was not performed as the number of included studies was below the recommended threshold [17].

## 3 Results

### 3.1 Study Selection and Characteristics

**Figure 1:**
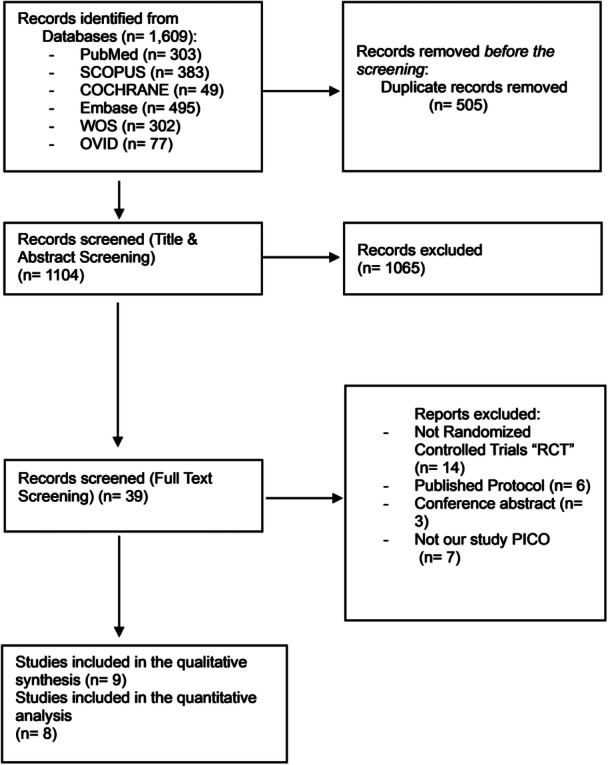
PRISMA flow diagram of the literature search.

A comprehensive literature search identified 1609 articles, of which 505 were duplicates. Following a two‐stage screening process—title and abstract screening, followed by full‐text review—nine RCTs [7, 8, 18, 19, 20, 21, 22, 23, 24] met the inclusion criteria and were included (Figure 1). One study, Wendt et al. [7], was excluded from the meta‐analysis due to the absence of relevant outcome data. The analyzed trials comprised a total of 11 589 patients: 5943 in the CEPD group and 5646 in the no‐CEPD group. Detailed characteristics of the patients and included studies are provided in Table 1 and Supporting Information S1: Table 2, respectively.

**Table 1:** Patients' baseline characteristics.

Authors' name, year, trial name	Study arms	Sample size of each arm	Age, mean ± SD, year	Gender, female (%)	BMI, kg/m^2^	NYHA classification, frequency (%)		STS PROM estimate, mean (SD), %	Past‐medical history									
						III	IV		AF, N (%)	Diabetes, *N* (%)	HTN, *N* (%)	PVD, *N* (%)	Prior stroke or TIA	CAD, *N* (%)	Previous MI, *N* (%)	Previous PCI	CHF	Previous CABG
Kharbanda et al. [8] [BHF PROTECT‐TAVI]	CEPD	3798	81.2 ± 6.5	1484 (39.1)	N/R	N/R	N/R	N/R	1256 (33.5)	793 (20.9)	2558 (68.4)	262 (7.7)	536 (14.3)	1234 (34.6)	N/R	N/R	N/R	N/R
	No CEPD	3803	81.3 ± 6.5	1461 (38.4)	N/R	N/R	N/R	N/R	1269 (33.8)	767 (20.2)	2528 (67.4)	255 (7.5)	526 (14.1)	1168 (32.9)	N/R	N/R	N/R	N/R
Kapadia et al. [20] [PROTECTED TAVR]	CEPD	1501	78.9 ± 8.0	631 (42)	N/R	N/R	N/R	3.3 ± 2.7	511 (34.1)	501 (33.4)	1306 (87.1)	165 (11.1)	114 (7.6)	850 (56.9)	N/R	495 (33.1)	N/R	N/R
	No CEPD	1499	78.9 ± 7.8	566 (37.8)	N/R	N/R	N/R	3.4 ± 2.8	469 (31.4)	522 (34.8)	1312 (87.6)	162 (10.9)	122 (8.2)	880 (58.9)	N/R	548 (36.6)	N/R	N/R
Lansky et al. [22] [REFLECT I]	CEPD	141	79.8 ± 7.3	N/R	N/R	96 (69.6)	4.6 ± 2.8	45 (33.1)	60 (42.9)	N/R	15 (11.2)	18 (13.1)	22 (17.1)	N/R	34 (26.2)	48 (35.6)	29 (21.5)	
	No CEPD	63	81.5 ± 7.1	N/R	N/R	49 (77.8)	4.8 ± 3.1	16 (25.8)	20 (31.7)	N/R	8 (13.6)	7 (11.3)	6 (10.2)	N/R	18 (30.0)	23 (37.1)	10 (16.1)	
Nazif et al. [23], [REFLECT II]	CEPD	157	80.31 ± 7.73	N/R	71 (45.2)	82 (52.5)	4.64 ± 2.77	44 (28)	61 (39.1)	N/R	20 (12.9)	27 (17.2)	30 (19.9)	N/R	49 (31.2)	86 (54.8)	29 (18.5)	
	No CEPD	57	78.05 ± 8.19		22 (38.6)	32 (56.2)	4.54 ± 2.50	17 (29.8)	23 (40.4)	N/R	11 (19.3)	3 (5.3)	13 (23.2)	N/R	15 (26.3)	33 (58.9)	11 (19.3)	
Kapadia et al. [19] [SENTINEL]	CEPD	121	83.1 (77.2–87.2)^a^	63 (52)	27.0 (23.7–32.1)^a^	101 (84.9)	5.6 (3.9–8.0)^a^	42 (34.7)	49 (40.5)	N/R	17 (14.0)	11 (11.5)	61 (50.4)	N/R	21 (17.4)	N/R	22 (18.2)	
	No CEPD	119	85 (78.4–89.4)^a^	58 (48.7)	27.0 (23.8–30.5)^a^	96 (82.8)	6.6 (4.5–8.6)^a^	36 (30.3)	45 (37.8)	N/R	18 (15.1)	14 (11.7)	66 (55.5)	N/R	20 (16.8)	N/R	25 (21.0)	
Haussig et al. [18], [CLEAN‐TAVI]	CEPD	50	80.0 ± 5.1	29 (58)	N/R	23 (46)	9 (18)	5.6 ± 3.2	N/R	20 (40)	44 (88)	2 (4)	1 (2)	26 (52)	6 (12)	5 (10)	46 (92)	8 (16)
	No CEPD	50	79.3 ± 4.1	28 (56)	N/R	29 (58)	3 (6)	5.2 ± 2.7	N/R	25 (50)	47 (94)	4 (8)	3 (6)	25 (50)	4 (8)	8 (16)	46 (92)	2 (4)
Van Mieghem et al. [6], [MISTRAL‐C]	CEPD	32	82 (79−84)^a^	15 (47)	N/R	18 (72%)	2 (8%)	4.6 (3.4−6.3)^a^	8 (29%)	4 (13%)	21 (66%)	9 (28%)	6 (19)	N/R	2 (6%)	N/R	N/R	N/R
	No CEPD	33	82 (77−86)	16 (49)	N/R	19 (66%)	4 (14%)	5.8 (3.5‐9.8)^a^	8 (27%)	9 (27%)	23 (70%)	11 (33%)	6 (18)	N/R	2 (6%)	N/R	N/R	N/R
Lansky et al. [21], [DEFLECT III]	CEPD	46	82.5 ± 6.5	56.50%	N/R	45.40%	6.3 ± 5.8	21.70%	21.70%	80.40%	13.00%	13.30%	N/R	13.00%	30.40%	N/R	10.90%	
	No CEPD	39	82.3 ± 6.0	51.30%	N/R	38.50%	7.4 ± 5.5	35.90%	23.10%	71.80%	12.80%	17.90%	N/R	21.10%	46.20%	N/R	7.70%	
Wendt et al. [7], [−]	CEPD	14	81.0 ± 5.0	10 (71.4)	N/R	N/R	N/R	11.4 ± 6.9	N/R	N/R	N/R	N/R	1 (7.1)	N/R	N/R	N/R	N/R	N/R
	No CEPD	16	82.1 ± 4.1	8 (50.0)	N/R	N/R	N/R	9.3 ± 6.3	N/R	N/R	N/R	N/R	3 (12.5)	N/R	N/R	N/R	N/R	N/R

Abbreviations: AF, atrial fibrillation; CAD, coronary artery diseases; CHF, congestive heart failure; HTN, hypertension; N/R, not reported; NYHA, New York Heart Association; PCI, Percutaneous coronary intervention; PVD, peripheral vascular diseases; STS PROM, Society of Thoracic Surgeons Predicted Risk of Mortality; TIA, Transient ischemic attack.

^a^ Values are median and interquartile range (IQR).

### 3.2 Studies' Quality Assessment

The trials demonstrated a low risk of bias. Where minor methodological limitations existed, reflecting the inherent open‐label nature of CEPD deployment and the inability to blind operators. All included studies were judged to have a low risk of bias, indicating overall high‐quality evidence—except for Wendt et al. [7], which was prematurely terminated, was rated to have a high risk of bias. Detailed information on the quality assessment is provided in Supporting Information S1: Figure 1.

### 3.3 Primary Outcomes

#### 3.3.1 Stroke

**Figure 2:**
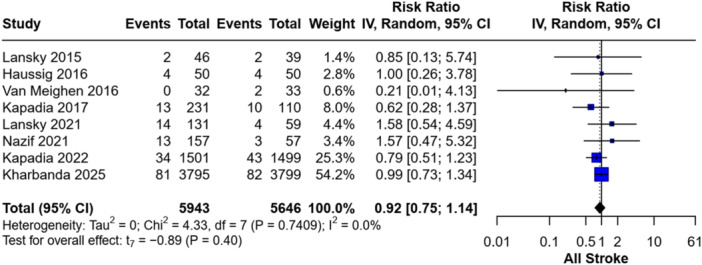
Forest plot of the incidence of all stroke events.

Eight RCTs reported the overall incidence of stroke following the TAVI procedure. We found no significant difference in stroke incidence between patients who underwent TAVI with CEPDs (*n* = 5943) and those without CEPDs (*n* = 5646), with a RR of 0.92 (95% CI: 0.75–1.14; *p* = 0.40). The included studies were homogeneous (*p* = 0.74; *I*² = 0%) (Figure 2).

**Figure 3:**
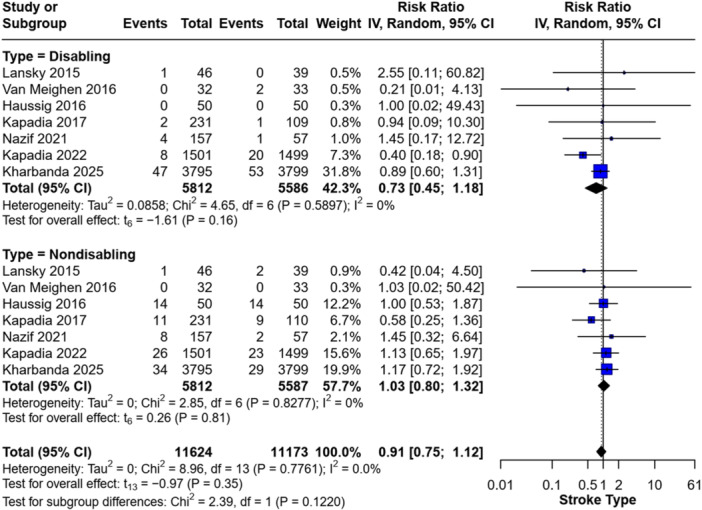
Forest plot of the incidence of disabling and non‐disabling strokes.

Subgroup analysis based on stroke type showed that CEPDs did not significantly reduce the risk of either disabling or non‐disabling stroke. The RR for non‐disabling stroke was 1.03 (95% CI: 0.80–1.32; *p* = 0.81), and for disabling stroke, it was 0.73 (95% CI: 0.45–1.18; *p* = 0.16) (Figure 3).

**Figure 4:**
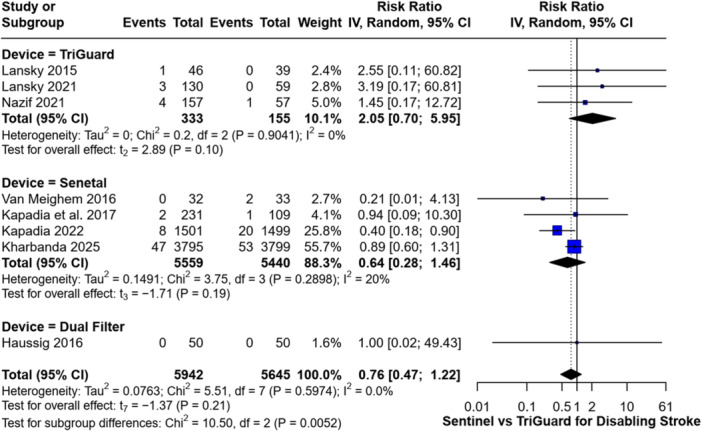
Forest plot comparing the incidence of disabling strokes between Sentinel and TriGuard devices.

Further analysis by device type and stroke subtype revealed that the Sentinel device showed a non‐statistically significant effect on disabling stroke, with an RR of 0.64 (95% CI: 0.28–1.46, *p* = 0.19). The TriGuard device demonstrated a non‐significant directional increase in the risk of disabling stroke, with an RR of 2.05 (95% CI: 0.70–5.95; *p* = 0.10) (Figure 4).

**Figure 5:**
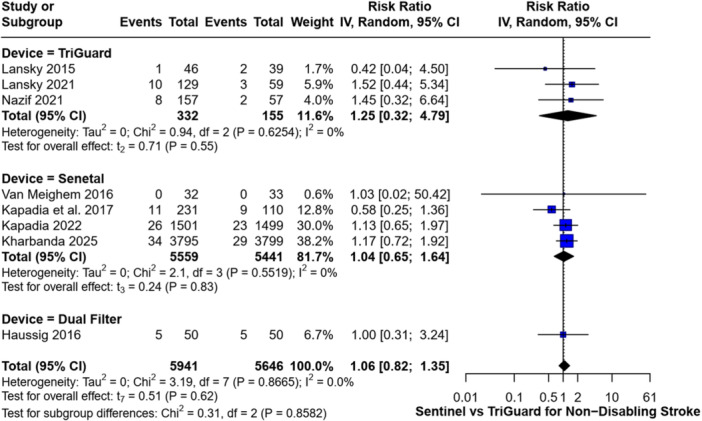
Forest plot comparing the incidence of non‐disabling strokes between Sentinel and TriGuard devices.

Similarly, there was no significant difference in the incidence of non‐disabling stroke between any of the devices and their respective control groups. The RR was 1.25 (95% CI: 0.32–4.79, *p* = 0.55) for Triguard, 1.04 (95% CI: 0.65–1.64, *p* = 0.83) for Sentinel, and 1.00 (95% CI: 0.31–3.24) for the Dual Filter System (Figure 5).

We also evaluated the incidence of stroke according to the timing of occurrence during follow‐up. No statistically significant differences were observed between the CEPD and non‐CEPD groups at 2–5 days (RR = 0.94; 95% CI: 0.75–1.19, *p* = 0.52), 30 days (RR = 0.86; 95% CI: 0.33–2.22, *p* = 0.68), or 90 days (RR = 0.95; 95% CI: 0.76–1.18) (Supporting Information S1: Figure 2).

##### 3.3.1.1 Mortality

**Figure 6:**
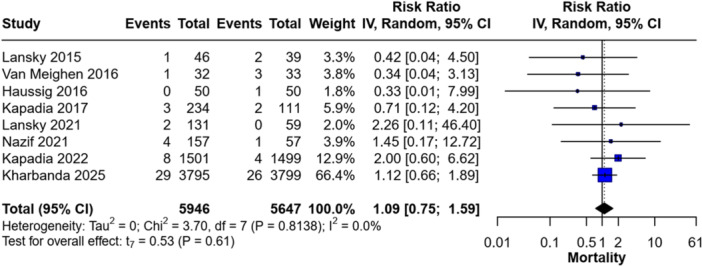
Forest plot of the incidence of all‐cause mortality.

No significant difference was observed in all‐cause mortality between the CEPD and non‐CEPD groups (RR = 1.09; 95% CI [0.75–1.59]; *p* = 0.61). The included studies demonstrated homogeneity (*I*² = 0%; *p* = 0.81) (Figure 6).

**Figure 7:**
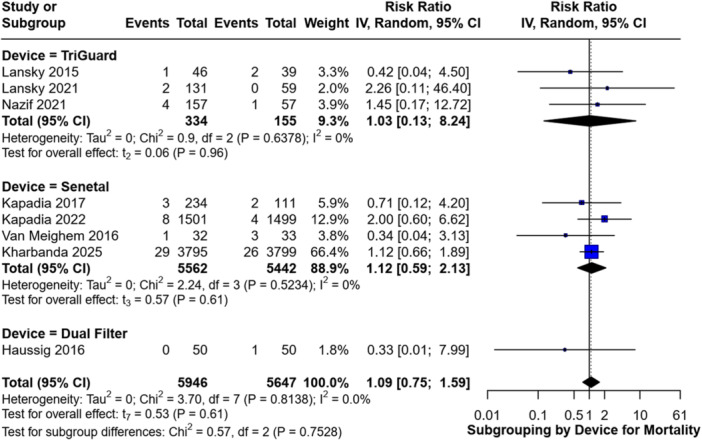
Forest plot comparing the incidence of all‐cause mortality between Sentinel and TriGuard devices.

Subgroup analysis by device type revealed no statistically significant differences compared to controls. For the TriGuard subgroup, the RR was 1.03 (95% CI [0.13–8.24], for the Sentinel subgroup, the RR was 1.12 (95% CI [0.59–2.13], and for the Dual Filter System subgroup, the RR was 0.33 (95% CI [0.01–7.99] (Figure 7).

##### 3.3.1.2 TSA of Primary Outcomes

**Figure 8:**
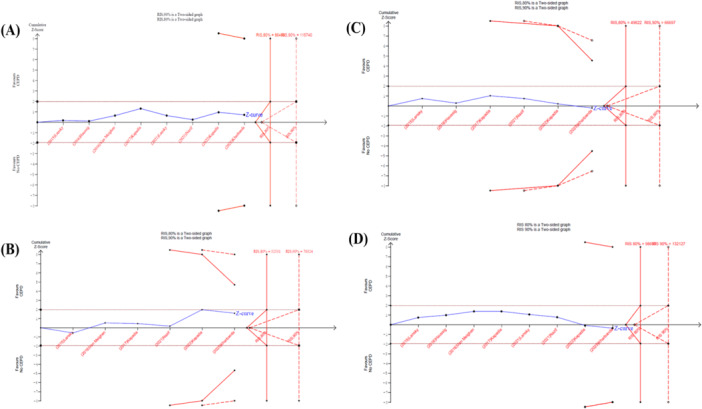
Trial Sequential Analysis (TSA) for the primary endpoints: (A) All Stroke, (B) Disabling Stroke, (C) Non‐Disabling Stroke, and (D) Mortality. TSA core assumptions: two‐sided alpha = 0.05, power = 80%, relative risk reduction = 20%, diversity‐adjusted RIS (*D*² applied), O'Brien‐Fleming boundaries.

A TSA was performed for the primary outcomes (disabling stroke, non‐disabling stroke, and all‐cause mortality). For all endpoints, the cumulative *Z*‐curve remained within the conventional significance boundaries and did not cross the trial sequential monitoring boundaries for benefit, harm, or futility. Specifically, the cumulative sample sizes did not reach the diversity‐adjusted RIS for any outcome (e.g., for disabling stroke, cumulative *n* = 11 589 vs. RIS = 52 531 [80% power] or 70 324 [90% power]; for all‐cause mortality, cumulative *n* = 11 589 vs. RIS = 98 667 [80% power] or 132 127 [90% power]) (Figure 8).

##### 3.3.1.3 Secondary Outcomes

###### 3.3.1.3.1 TIA

Three studies reported the incidence of TIAs. The meta‐analysis showed no significant difference between the CEPD and non‐CEPD groups (RR = 1.28; 95% CI: 0.56–2.95; *p* = 0.33), with no heterogeneity observed (*I*² = 0%; *p* = 0.72). (Supporting Information S1: Figure 3).

##### 3.3.1.4 Life‐Threatening or Disabling Bleeding

Six trials reported the incidence of life‐threatening or disabling bleeding. The overall analysis showed no significant difference in the risk of postprocedural life‐threatening bleeding between the CEPD and non‐CEPD groups (RR = 1.22; 95% CI: 0.59–2.52; *p* = 0.51), with low heterogeneity (*I*² = 14%; *p* = 0.32) (Supporting Information S1: Figure 4). Similarly, subgroup analysis by device type revealed no significant differences when comparing Triguard (RR = 1.82; *p* = 0.54) or Sentinel (RR = 0.70; *p* = 0.76) or Dual Filter System (RR = 1.22) with their controls (Supporting Information S1: Figure 5).

##### 3.3.1.5 MVCs

Six trials reported the incidence of postprocedural MVCs, defined according to VARC criteria. Notably, the two largest trials (BHF PROTECT‐TAVI and PROTECTED TAVR) did not systematically report this endpoint, resulting in a restricted pooled sample size. The meta‐analysis revealed no significant difference in the incidence of postprocedural MVCs between the CEPD and non‐CEPD groups (RR = 1.17; 95% CI: 0.47–2.91; *p* = 0.67), with moderate heterogeneity (*I*² = 48%; *p* = 0.08) (Supporting Information S1: Figure 6). For subgroups based on device type, no statistically significant differences were observed with the Triguard device (RR = 2.69; *p* = 0.33), the Sentinel device (RR = 0.47; *p* = 0.69), or Dual Filter System (RR = 0.83) (Supporting Information S1: Figure 7).

##### 3.3.1.6 NIHSS Score Worsening

No significant differences were observed in NIHSS scores between the CEPD and control groups at various follow‐up intervals. At 2–5 days, the RR was 0.98. (95% CI: 0.42–2.30), *p* = 0.96; at 30 days, RR = 1.49 (95% CI 0.75–2.94), *p* = 0.16; and at 90 days, RR = 1.23 (95% CI: 0.13–11.48). The overall pooled analysis also showed no significant difference between groups (RR = 1.12; 95% CI: 0.76–1.65; *p* = 0.54), with low heterogeneity across studies (*I*² = 5.5%; *p* = 0.39) (Supporting Information S1: Figure 8).

##### 3.3.1.7 MoCA Score Worsening

MoCA scores were reported in three studies. At 2–5 days of follow‐up, patients in the CEPD group exhibited significantly less reduction in MoCA scores compared to controls (RR = 0.71; 95% CI: 0.59–0.85). However, no significant differences were observed at 30 days (RR = 1.11; 95% CI: 0.50–2.44) or 90 days (RR = 1.07; 95% CI: 0.30–3.81). The overall pooled analysis did not demonstrate a significant difference between the CEPD and control groups (RR = 0.84; 95% CI: 0.64–1.10; *p* = 0.17), with no observed heterogeneity (*I*² = 0%; *p* = 0.54) (Supporting Information S1: Figure 9).

##### 3.3.1.8 Aki

The overall analysis revealed no significant difference in the risk of AKI between the CEPD and non‐CEPD groups (RR = 1.18; 95% CI: 0.95–1.48; *p* = 0.11), with no significant heterogeneity across studies (*I*² = 0%; *p* = 0.66) (Supporting Information S1: Figure 10). Subgroup analysis by device type also showed no significant differences in AKI risk when comparing Triguard (RR = 1.91; 95% CI: 0.17–20.99) or Sentinel (RR = 1.20; 95% CI: 1.01–1.44) or Dual Filter System (RR = 0.20; 95% CI: 0.02–1.65) devices with their respective control groups (Supporting Information S1: Figure 11).

##### 3.3.1.9 Delirium

Two studies reported the incidence of post‐procedural delirium. The pooled analysis showed no significant difference between the CEPD and control groups (RR = 0.63; *p* = 0.66) (Supporting Information S1: Figure 12).

##### 3.3.1.10 Pacemaker Implantation

Three studies reported the need for pacemaker implantation. The pooled results indicated no significant difference between patients who received CEPDs and those who did not (RR = 1.09; 95% CI: 0.88–1.36; *p* = 0.22), with no observed heterogeneity (*I*² = 0%; *p* = 0.63) (Supporting Information S1: Figure 13).

##### 3.3.1.11 GRADE Assessment

The outcomes were evaluated using the GRADE criteria, and all were rated as having low certainty of evidence. A detailed summary of findings, along with the certainty ratings, is presented in Supporting Information S1: Table 3.

## 4 Discussion

During TAVI, the embolic debris generated during valve preparation, deployment, and manipulation is a major contributor to these neurological events. CEPDs were developed to mitigate the risk of stroke by capturing debris during valve deployment. While the mechanistic rationale for CEPDs is compelling, their clinical utility remains debated. This meta‐analysis synthesized the current evidence from eight RCTs to provide the most comprehensive evaluation to date on the impact of CEPD use on clinical outcomes.

Our primary analysis revealed no statistically significant difference in the overall incidence of stroke between the CEPD and non‐CEPD groups. This lack of difference extended to subgroup analyses based on stroke severity, with no significant reduction observed for either disabling stroke or non‐disabling stroke. Our analysis found no significant difference in all‐cause mortality between patients treated with and without CEPDs. Subgroup analyses based on specific CEPD types (Sentinel, Triguard, Dual Filter System) did not reveal any significant reduction in mortality compared to controls.

Regarding secondary outcomes, we observed no significant differences between CEPD and non‐CEPD groups for TIA, life‐threatening/disabling bleeding, MVCs, AKI, delirium, or new pacemaker implantation. Notably, CEPDs were associated with a transient improvement in MoCA scores at 2–5 days, though this did not persist at 30 or 90 days, and the overall pooled analysis for MoCA worsening was non‐significant. TSA indicated insufficient evidence to confirm futility, suggesting larger trials may still be warranted to definitively refute a clinically meaningful benefit or harm of CEPDs for these endpoints.

We sub‐grouped outcomes based on the type of CEPD used, which varied across the included studies. The TriGuard device was employed in the DEFLECT III, REFLECT I, and REFLECT II trials [21, 22, 23]. These devices are positioned in the aortic arch and are designed to cover the innominate, left common carotid, and subclavian arteries throughout the TAVI procedure. The Claret Montage Dual Filter System was used in the CLEAN‐TAVI trial [18], while the SENTINEL device was used in the BHF PROTECT‐TAVI, MISTRAL‐C, SENTINEL, and PROTECTED TAVR trials [8, 19, 20, 24]. Both the Sentinel and Dual Filter systems are deployed in the innominate artery and the left common carotid artery, with the Sentinel featuring an updated ergonomic handle and extended catheter length compared to earlier designs [25].

A previous meta‐analysis by Pérez‐Camargo et al. [26] reported no significant difference in 30‐day stroke rates between the two types of CEPDs: TriGuard (*p* = 0.600) versus Sentinel (*p* = 0.064). Another meta‐analysis conducted by Harmouch et al. [27] focused on the Sentinel device and included three trials: MISTRAL‐C, SENTINEL, and PROTECTED TAVR, as well as the CLEAN‐TAVI trial for the Dual Filter system. While they did not differentiate between the Dual Filter and Sentinel devices as our analysis, they similarly found no difference in overall periprocedural stroke incidence between TAVI with and without the Sentinel device. Notably, they observed a statistically significant reduction in disabling stroke with the Sentinel (RR 0.41; 95% CI: 0.20–0.86; *p* = 0.02). In our analysis—which includes the more recent BHF PROTECT‐TAVI trial within the Sentinel subgroup—the findings remain aligned with those of Harmouch et al. [27] regarding other outcomes, including all‐cause mortality and TIAs.

Importantly, CEPDs should not be viewed as a homogeneous intervention. These distinct operational profiles intrinsically dictate varying safety and efficacy considerations. Our conservative statistical framework did not yield a statistically significant increase in MVCs for the TriGuard system. But, the requirement for an additional transfemoral sheath and extensive arch navigation with deflector devices may interact differently with tortuous or atherosclerotic anatomy, potentially predisposing certain patients to access‐site or arch‐related complications. Therefore, the procedural decision‐making should account for individual patient anatomy when weighing the risks and benefits of specific device platforms.

Previous meta‐analyses by Basit et al. [28] and Baloch et al. [29] reported a reduction in overall stroke incidence among patients receiving CEPDs. However, this benefit was primarily driven by observational studies. When the analyses were stratified by study design, the significance was lost in the RCT subgroup and persisted only in the observational evidence. Although large registry‐based observational studies may suggest a potential benefit, their findings should be interpreted with caution due to inherent limitations, including residual confounding—even after propensity score matching. Notably, even with a cumulative sample of 414 649 patients, the observed reduction in stroke events was marginal and of borderline statistical significance, reinforcing the importance of RCTs as a more robust and unbiased source of evidence [27].

The mechanical interaction between transcatheter equipment and the calcified aortic valve complex (specifically during balloon predilatation and postdilatation) is a known driver of periprocedural embolization. In our analysis, we noted that while procedural dilatation rates varied among the included trials, they were well‐balanced between the CEPD and control arms in the largest pivotal studies (BHF PROTECT‐TAVI and PROTECTED TAVR), Supporting Information S1: Table 5. Subgroup analyses from these trials demonstrated that the presence or absence of balloon dilatation did not significantly interact with the overall efficacy of CEPD regarding total stroke incidence.

The transient improvement observed in early MoCA scores (less worsening at 2−5 days) is intriguing and echoes findings from some individual trials suggesting potential benefits on subclinical or subtle neurological injury. This also aligns with our previous meta‐analysis on 4091 patients [30]. However, the modest early improvements observed in neurocognitive screening should be interpreted as surrogate findings rather than evidence of definitive clinical benefit, particularly because our primary analysis did not demonstrate a significant reduction in overall, disabling, or non‐disabling stroke or mortality with systematic CEPD use. Although several mechanistic trials that incorporated routine diffusion‐weighted MRI reported reductions in lesion burden and occasional short‐term signals favoring early cognitive preservation, these effects were not consistently sustained at later follow‐up and were not accompanied by parallel improvements in hard clinical outcomes.

The TSA results are particularly crucial, emphasizing that while current evidence does not support a significant benefit for stroke or mortality reduction, it is also insufficient to definitively rule out a modest effect, underscoring the need for ongoing evaluation or potentially larger datasets. Our TSA findings are generally consistent with those of Warraich et al. [31], whose analysis included seven of the trials used in our study, but didn't include the BHF PROTECT‐TAVI trial. In both cases, the TSA for all stroke and disabling stroke remained within the non‐statistically significant zone, with our TSA showing that the *Z*‐curve shows a greater extension, approaching the futility boundary. However, a key difference emerged in the TSA of all‐cause mortality: our *Z*‐curve did not cross into the futility zone, in contrast to the findings of Warraich et al. despite our total sample size being more than twice as large. This discrepancy is attributable to differences in the assumed relative risk reduction (RRR) used in RIS calculations. Warraich et al. [31] applied a RRR of 44.06% for all‐cause mortality, whereas we opted for a more clinically meaningful and conservative RRR of 20%, which is a more conservative and realistic effect size [32].

## 5 Clinical Implications, Limitations, and Future Recommendations

High‐risk patient characteristics include heavily calcified bicuspid aortic valves, severe aortic arch atheroma, and a prior history of cerebrovascular events [25]. The embolic burden may further increase with procedural factors as aggressive balloon pre‐ or post‐dilatation, repeated valve repositioning, and valve‐in‐valve interventions. In these contexts, the neuroprotective effect of CEPDs may be more pronounced. This potential benefit must be balanced against device‐related risks. Patients with severe peripheral arterial disease, marked vascular tortuosity, or complex aortic arch anatomy are at increased risk of access‐site and MVCs. Therefore, CEPD use should be individualized.

Several limitations should be considered when interpreting the findings of this meta‐analysis. Although we included only RCTs, moderate heterogeneity (*I*² = 48%) was observed for the outcome of MVCs, likely reflecting variability across studies—potentially due to differences in device types (as highlighted in our subgroup analysis), baseline patient characteristics (e.g., vascular access anatomy), procedural techniques, or variations in endpoint definitions. In contrast, heterogeneity for stroke and mortality outcomes was low (*I*² = 0%), though subtle inter‐trial differences may still exist. Additionally, pooling data from various CEPD types—primarily Sentinel, TriGuard, and the Dual Filter System—may have obscured device‐specific efficacy and safety signals. While subgroup analyses were conducted to explore this, limited sample sizes for some devices (e.g., the Dual Filter System) reduced the statistical power of these comparisons. For neurocognitive outcomes (MoCA and NIHSS), analysis using continuous mean differences is generally preferred. However, this approach was not feasible because several key trials did not report the necessary variance measures. Instead, these studies reported outcomes categorically as the proportion of patients experiencing a “worsening” event, trial‐specific thresholds. For example, the REFLECT I trial strictly defined MoCA worsening as a decrease of 3 or more points from baseline, and NIHSS worsening as any clinically relevant positive increment determined by a blinded neurologist. Because these explicit thresholds and the rigor of assessor blinding were not uniformly standardized across all included studies, we acknowledge that our reliance on dichotomized pooling introduces heterogeneity and may limit the sensitivity to detect subtle continuous shifts.

Our findings, in line with the TSA, highlight the need for additional high‐quality evidence before drawing firm conclusions about CEPD efficacy—particularly for disabling stroke. Future studies should include larger RCTs, possibly enriched with higher‐risk populations, where potential benefits may be more discernible. Given the potential for device‐specific effects—especially the observed safety signal of increased MVCs with TriGuard—further evaluation of individual CEPD performance is warranted. This includes head‐to‐head RCTs, detailed registries, and mechanistic studies to explore the cause of adverse events associated with certain devices. Future trials should also adopt standardized neurological assessments, incorporate more sensitive neurocognitive tools beyond MoCA, and utilize advanced imaging modalities such as MRI to better capture clinical and subclinical brain injury.

## 6 Conclusions

In the largest meta‐analysis of randomized trial data on CEPDs during TAVI, no device demonstrated a clinically meaningful reduction in overall stroke or mortality. This was consistent through subgroup analyses based on stroke or device types. TSA suggested that current evidence remains inconclusive. There is a need for further high‐powered RCTs, particularly in patients at elevated embolic risk.

## Author Contributions

**Mohamed Ibrahim Gbreel** and **Ahmed Samy Badran:** conceptualization, methodology, software, formal analysis, project administration, writing – original draft preparation. **Marwa Hassan:** conceptualization, methodology, writing – original draft, writing – review and editing, visualization. **Mahmoud Balata:** conceptualization, methodology, writing – original draft, writing – review and editing, supervision.

## Funding

The authors have nothing to report.

## Ethics Statement

The authors have nothing to report.

## Conflicts of Interest

The authors declare no conflicts of interest.

## Supporting information

Supporting File

## Data Availability

All data generated or analyzed during this study are included in this published article. All additional raw data is available upon request from the corresponding author.
