# Supplementary material for: Cerebral Embolic Protection Devices (CEPDs) During Transcatheter Aortic Valve Implantation (TAVI): A Meta‐Analysis of Randomized Controlled Trials
Source: Clin Cardiol. 2026 Apr 23;49(4):e70309. doi: 10.1002/clc.70309 (PMC13103726; doi:10.1002/clc.70309)
Supplement: Supplementary file 1 — Supporting File [file CLC-49-e70309-s001.docx]

**Supplementary Materials**

**Supplementary Table 1.** Search Strategy for each database.

**Supplementary Table 2.** Summary of the included studies.

**Supplementary Table 3.** Summary of findings table, GRADE assessment.

**Supplementary Table 4.** Stroke definitions and adjudication methods across included trials

**Supplementary Table 5.** Rates of balloon Predilatation and Postdilatation methods across included trials

**Supplementary Figure 1:** Risk of bias assessment of randomized controlled trials.

**Supplementary Figure 2:** Forest plot of stroke incidence according to the timing of occurrence during follow-up.

**Supplementary Figure 3:** Forest plot of incidence of transient ischemic attacks (TIAs).

**Supplementary Figure 4:** Forest plot of incidence of life-threatening or disabling bleeding.

**Supplementary Figure 5:** Forest plot of subgroup analysis of the incidence of life-threatening or disabling bleeding by device.

**Supplementary Figure 6:** Forest plot of the incidence of Major vascular complications.

**Supplementary Figure 7:** Forest plot of subgroup analysis of the incidence of Major vascular complications by device.

**Supplementary Figure 8:** Forest plot of National Institutes of Health Stroke Scale (NIHSS) Score Worsening.

**Supplementary Figure 9:** Forest plot of Montreal Cognitive Assessment (MoCA) Score Worsening.

**Supplementary Figure 10:** Forest plot of the incidence of Acute Kidney Injury (AKI).

**Supplementary Figure 11:** Forest plot of subgroup analysis of the incidence of Acute Kidney Injury (AKI) by device**.**

**Supplementary Figure 12:** Forest plot of the incidence of post-procedural delirium.

**Supplementary Figure 13:** Forest plot of the need for pacemaker implantation.

**PRISMA-checklist**

**Supplementary Table 1.** Search Strategy for each database.

| **Database** | **Strategy** |
| --- | --- |
| **PubMed** | ("Transcatheter Aortic Valve Implantation"[tiab] OR "TAVI"[tiab] OR "Transcatheter Aortic Valve Replacement"[tiab] OR "TAVR"[tiab] OR "Percutaneous Aortic Valve Replacement"[tiab] OR "Percutaneous Aortic Valve Implantation"[tiab] OR "Catheter-based Aortic Valve Implantation"[tiab] OR "Catheter-based Aortic Valve Replacement"[tiab] OR "Aortic Valve Transcatheter Implantation"[tiab] OR "Aortic Valve Transcatheter Replacement"[tiab] OR "Transcatheter Aortic Valve Replacement"[Mesh]) AND ("Embolic Protection Device"[tiab] OR "Embolic Protection Devices"[tiab] OR "EPD"[tiab] OR "Cerebral protection"[tiab] OR "Cerebral Embolic protection device"[tiab] OR "Cerebral Embolic protection devices"[tiab] OR "CEPD"[tiab]) |
| **Web of Science (WOS)** | TS=("Transcatheter Aortic Valve Implantation" OR "TAVI" OR "Transcatheter Aortic Valve Replacement" OR "TAVR" OR "Percutaneous Aortic Valve Replacement" OR "Percutaneous Aortic Valve Implantation" OR "Catheter-based Aortic Valve Implantation" OR "Catheter-based Aortic Valve Replacement" OR "Aortic Valve Transcatheter Implantation" OR "Aortic Valve Transcatheter Replacement") AND TS=("Embolic Protection Device" OR "Embolic Protection Devices" OR "EPD" OR "Cerebral protection" OR "Cerebral Embolic protection device" OR "Cerebral Embolic protection devices" OR "CEPD") |
| **Scopus** | TITLE-ABS-KEY ( ( "Transcatheter Aortic Valve Implantation" OR "TAVI" OR "Transcatheter Aortic Valve Replacement" OR "TAVR" OR "Percutaneous Aortic Valve Replacement" OR "Percutaneous Aortic Valve Implantation" OR "Catheter-based Aortic Valve Implantation" OR "Catheter-based Aortic Valve Replacement" OR "Aortic Valve Transcatheter Implantation" OR "Aortic Valve Transcatheter Replacement" ) AND ( "Embolic Protection Device" OR "Embolic Protection Devices" OR "EPD" OR "Cerebral protection" OR "Cerebral Embolic protection device" OR "Cerebral Embolic protection devices" OR "CEPD" ) ) |
| **Cochrane Library** | ("Transcatheter Aortic Valve Implantation" OR "TAVI" OR "Transcatheter Aortic Valve Replacement" OR "TAVR" OR "Percutaneous Aortic Valve Replacement" OR "Percutaneous Aortic Valve Implantation" OR "Catheter-based Aortic Valve Implantation" OR "Catheter-based Aortic Valve Replacement" OR "Aortic Valve Transcatheter Implantation" OR "Aortic Valve Transcatheter Replacement"):ti,ab,kw AND ("Embolic Protection Device" OR "Embolic Protection Devices" OR "EPD" OR "Cerebral protection" OR "Cerebral Embolic protection device" OR "Cerebral Embolic protection devices" OR "CEPD"):ti,ab,kw |
| **Embase** | ('transcatheter aortic valve implantation'/exp OR 'transcatheter aortic valve implantation':ti,ab OR 'tavi':ti,ab OR 'transcatheter aortic valve replacement'/exp OR 'transcatheter aortic valve replacement':ti,ab OR 'tavr':ti,ab OR 'percutaneous aortic valve replacement':ti,ab OR 'percutaneous aortic valve implantation':ti,ab OR 'catheter-based aortic valve implantation':ti,ab OR 'catheter-based aortic valve replacement':ti,ab) AND ('embolic protection device'/exp OR 'embolic protection device':ti,ab OR 'embolic protection devices':ti,ab OR 'epd':ti,ab OR 'cerebral protection'/exp OR 'cerebral protection':ti,ab OR 'cerebral embolic protection device':ti,ab OR 'cerebral embolic protection devices':ti,ab OR 'cepd':ti,ab) |
| **Ovid MEDLINE** | (Transcatheter Aortic Valve Implantation.ti,ab. OR TAVI.ti,ab. OR Transcatheter Aortic Valve Replacement.ti,ab. OR TAVR.ti,ab. OR Percutaneous Aortic Valve Replacement.ti,ab. OR Percutaneous Aortic Valve Implantation.ti,ab. OR Catheter-based Aortic Valve Implantation.ti,ab. OR Catheter-based Aortic Valve Replacement.ti,ab. OR Aortic Valve Transcatheter Implantation.ti,ab. OR Aortic Valve Transcatheter Replacement.ti,ab. OR exp Transcatheter Aortic Valve Replacement/) AND (Embolic Protection Device.ti,ab. OR Embolic Protection Devices.ti,ab. OR EPD.ti,ab. OR Cerebral protection.ti,ab. OR Cerebral Embolic protection device.ti,ab. OR Cerebral Embolic protection devices.ti,ab. OR CEPD.ti,ab.) |

| **Supplementary Table 2.** Summary of the included studies | | | | | | | | | |  |  |
| --- | --- | --- | --- | --- | --- | --- | --- | --- | --- | --- | --- |
| **Authors' name, Year, Trial name** | **Design** | **NCT Number** | **Setting** | **Sample Size** | **Stroke definition** | **Valve replaced** | **Cerebral Protection Device** | **Follow-up periods** |  |  |  |
| **Kharbanda et al., 2025, [BHF PROTECT-TAVI]** | Multicenter, RCT | ISRCTN16665769 | UK | 7635 | VARC-2 | Self-expanding valve and Balloon-expanding valve | Sentinel, Boston Scientific | The 72 hours after TAVI |  |  |  |
| **Kapadia et al., 2022, [PROTECTED TAVR]** | Multicenter, RCT | NCT04149535 | North America, Europe, and Australia | 3000 | VARC-2 | Balloon-expandable and non-balloon expandable. | The Sentinel cerebral embolic protection device (Boston Scientific) | 72 hours after TAVR or before discharge (whichever came first) and 30 days. |  |  |  |
|  |  |  |  |  |  |  |  |  |  | |  |
| **Lansky et al., 2021, [REFLECT I]** | Prospective, multicenter RCT | NCT02536196 | USA | 258 | VARC-2 | The commercial transcatheter valve systems, according to standard institutional procedures under local or general anaesthesia. | The TriGuard HDH embolic deflection device (Keystone Heart Ltd., | 2–5, 30, and 90 days following the procedure. |  | |  |
|  |  |  |  |  |  |  | Caesarea, IL, USA) |  |  | |  |
| **Nazif et al., 2021, [REFLECT II]** | Multicenter, RCT | NCT02536196 | USA | 220 | VARC-2 | The commercially available transcatheter valve systems. | The TriGuard HDH embolic deflection device (Keystone Heart Ltd., Caesarea, IL, USA) | Before discharge or at 2 to 5 days, and at 30 days, with phone follow-up for assessment of stroke and vital status at 90 days. |  | |  |
|  |  |  |  |  |  |  |  |  |  | |  |
| **Kapadia et al., 2017, [SENTINEL]** | Multicenter, RCT | NCT02214277 | USA and Germany | 363 | VARC-2 | SAPIEN XT (17.8%) and SAPIEN 3 | The Sentinel cerebral embolic protection device (Boston Scientific) | 2 to 7, 30, and 90 days. |  | |  |
|  |  |  |  |  |  | (52.4%) (Edwards Lifesciences, Irvine, California) |  |  |  | |  |
|  |  |  |  |  | VARC-2 | and CoreValve (3.9%) and Evolut R (25.9%) (Medtronic, Minneapolis, Minnesota). |  |  |  | |  |
| **Haussig et al., 2016, [CLEAN-TAVI]** | Single-center, RCT | NCT01833052 | Germany | 100 | VARC-2 | Medtronic CoreValve (Medtronic) self-expanding | Claret Montage Dual Filter System (Claret Medical Inc) | Day 2, 7, and 30: Following the procedure |  | |  |
| **Van Mieghem et al., 2016, [MISTRAL-C]** | Multicenter, RCT | NCT02214277 | USA and Germany | 65 | VARC-2 | Medtronic CoreValve, Edwards SAPIEN XT, Edwards SAPIEN 3, and Balloon dilatation | Sentinel™ Cerebral Protection System | 30 days |  | |  |
| **Lansky et al., 2015, [DEFLECT III]** | Multicenter RMulticenter, RCTT | NCT02070731 | European Union and Israel | 85 | VARC-2 | Edwards SAPIEN/XT/3, Medtronic CoreValve, and other commercial valves. | The TriGuard HDH embolic deflection device (Keystone Heart Ltd., Caesarea, IL, USA) | In-hospital (4±2 days), and 30±7 days following procedure. |  | |  |
|  |  |  |  |  |  |  |  |  |  | |  |
| **Wendt et al., 2015, [-]** | Single-center, RCT | NCT01735513 | Germany | 30 | - | SAPIEN XT prosthesis (Edwards Lifesciences) | Embol-X Trans-aortic | 1 month |  | |  |
| **RCT**: Randomized controlled trial | | | | | | | | | | | |

| **Supplementary Table 3.** Summary of findings table, GRADE assessment | | | | | | | | | | | | | |  |
| --- | --- | --- | --- | --- | --- | --- | --- | --- | --- | --- | --- | --- | --- | --- |
| **Outcome name** | **Number of included studies** | **Design of included studies** | **Effect size (95% CI)** | **Heterogeneity** | **Number of patients in the CEPD group** | **Number of patients in the non-CEPD group** | **Risk of bias** | **Inconsistency** | **Indirectness** | **Imprecision** | **Publication bias** | **Other considerations (a)** | **Quality of evidence** |  |
| **All Strokes** | 8 studies with 11589 patients | RCTs | 0.92 (95% CI: 0.75–1.14) | (P = 0.74; I² = 0%) | 5943 | 5646 | Not serious | Not serious | Not serious | Serious (b) | Uncertain (c) | Not existed | Low ⊕ ⊕ ◯ ◯ |  |
| **Disabling** | 8 studies with 11398 patients | RCTs | 0.73 (95% CI: 0.45–1.18) | (P = 0.46; I² = 0%) | 5812 | 5586 | Not serious | Not serious | Not serious | Serious (b) | Uncertain (c) | Not existed | Low ⊕ ⊕ ◯ ◯ |  |
| **Non-disabling stroke** | 8 studies with 11398 patients | RCTs | 1.03 (95% CI: 0.80–1.32) | (P = 0.72; I² = 0%) | 5812 | 5586 | Not serious | Not serious | Not serious | Serious (b) | Uncertain (c) | Not existed | Low ⊕ ⊕ ◯ ◯ |  |
| **All-Cause Mortality** | 8 studies with 11593 patients | RCTs | 1.09 (95% CI: 0.75–1.59) | (P = 0.81; I² = 0%) | 5946 | 5647 | Not serious | Not serious | Not serious | Serious (b) | Uncertain (c) | Not existed | Low ⊕ ⊕ ◯ ◯ |  |
| **Transient Ischemic Attack (TIA)** | 3 studies with 10935 patients | RCTs | 1.28 (95% CI: 0.56–2.95) | (P = 0.72; I² = 0%) | 5527 | 5408 | Not serious | Not serious | Not serious | Serious (b) | Uncertain (c) | Not existed | Low ⊕ ⊕ ◯ ◯ |  |
| **Life-threatening or disabling bleeding** | 6 studies with 8171 patients | RCTs | 1.22 (95% CI: 0.59–2.52) | (P = 0.32; I² = 14%) | 4176 | 3995 | Not serious | Not serious | Not serious | Serious (b) | Uncertain (c) | Not existed | Low ⊕ ⊕ ◯ ◯ |  |
| **Major vascular complications (MVCs)** | 6 studies with 1016 patients | RCTs | 1.17 (95% CI: 0.47–2.91) | (P = 0.08; I² = 48.5%) | 659 | 357 | Not serious | Not serious | Not serious | Serious (b) | Uncertain (c) | Not existed | Low ⊕ ⊕ ◯ ◯ |  |
| **RCTs:** randomized controlled trials, **CI:** confidence interval, **RR:** risk ratio (a) Other considerations are large effect, dose-response, and plausible confounding factors (b) As the analysis showed a wide confidence interval and Trial Sequential Analysis (TSA) demonstrated that the cumulative sample size has not met the Required Information Size (RIS), and boundaries for benefit or futility were not crossed.  (c) Not assessed due to <10 studies Moderate indicates that confidence in the result is moderate, but further research could have an important impact and might alter the estimated effect. | | | | | | | | | | | | | |  |
|  |  |  |  |  |  |  |  |  |  |  |  |  |  |  |
|  |  |  |  |  |  |  |  |  |  |  |  |  |  |  |

**Supplementary Table 4.** Stroke Definitions and Adjudication Methods Across Included Trials

| **Trial (Year)** | **Stroke Definition** | **Disabling Stroke Threshold** | **Extracted Timepoint** | **Adjudication Method** |
| --- | --- | --- | --- | --- |
| **BHF PROTECT-TAVI (2025)** | Clinical deficit lasting >24h (or mechanical thrombectomy) | mRS ≥ 2 (and ≥ 1 point increase from baseline) | ≤ 72 hours or hospital discharge (6-8 weeks for mRS) | Blinded independent CEC comprised of neurologists. Daily QVSFS screening used. |
| **PROTECTED TAVR (2022)** | NeuroARC criteria (clinical stroke, Types 1.a-d, 2.a) | mRS ≥ 2 (and ≥ 1 point increase from baseline) | ≤ 72 hours or hospital discharge (30 days for mRS) | Neurology professional assessment at baseline and post-TAVR; independent, blinded CEC adjudication. |
| **REFLECT II (2021)** | VARC-2 and NeuroARC definitions | VARC-2 criteria (disabling classification via independent CEC) | 30 days (safety and efficacy composite) / In-hospital (MACCE) | Blinded neurologist or clinical designee assessment; independent CEC adjudication. |
| **REFLECT I (2021)** | VARC-2 and NeuroARC criteria | VARC-2 criteria (via NIHSS and mRS) | 2-5 days, 30 days, and 90 days | Blinded board-certified neurologist assessment; independent CEC adjudication. |
| **SENTINEL (2017)** | VARC-2 criteria | VARC-2 criteria (Assessed via mRS and NIHSS at 90 days) | 30 days (initial occurrence) and 90 days (severity determination) | Formal assessment administered directly by a neurologist. |
| **MISTRAL-C (2016)** | VARC-2 criteria | VARC-2 criteria (Assessed via mRS and NIHSS) | 5 to 7 days (neurological exam) and 30 days (clinical endpoint reporting) | Assessment by a trained, blinded neurology specialist. |
| **CLEAN-TAVI (2016)** | VARC-2 criteria | VARC-2 criteria (via NIHSS and mRS) | 2 days, 7 days, and 30 days | Assessment by blinded, NIHSS/mRS-certified personnel (attending physician/scientist). |
| **DEFLECT III (2015)** | VARC-2 criteria (and new neurologic impairment via NIHSS + DW-MRI) | VARC-2 criteria (Assessed via NIHSS and mRS) | Pre-discharge (mean 6.2 days) and 30 days | Blinded, certified neurologic assessors; independent CEC adjudication (including a vascular neurologist). |

**CEC:** Clinical Events Committee, **DW-MRI:** Diffusion-Weighted Magnetic Resonance Imaging, **MACCE:** Major Adverse Cardiovascular and Cerebrovascular Events, **mRS:** Modified Rankin Scale, **NeuroARC:** Neurologic Academic Research Consortium, **NIHSS:** National Institutes of Health Stroke Scale, **QVSFS:** Questionnaire for Verifying Stroke-Free Status, **VARC-2:** Valve Academic Research Consortium-2

**Supplementary Table 5.** Rates of Balloon Predilatation and Postdilatation Across Included Trials

| **Trial (Year)** | **Predilatation (CEP Group)** | **Predilatation (Control Group)** | **Postdilatation (CEP Group)** | **Postdilatation (Control Group)** |
| --- | --- | --- | --- | --- |
| **BHF PROTECT-TAVI (2025)** | 57.3% (2,166 / 3,777) | 57.3% (2,167 / 3,781) | 45.2% (1,714 / 3,788) | 45.3% (1,718 / 3,793) |
| **PROTECTED TAVR (2022)** | 38.5% (573 / 1,489) | 41.9% (624 / 1,490) | 26.2% (390 / 1,489) | 25.7% (383 / 1,490) |
| **REFLECT II (2021)** | 24.1% (27 / 112)* | 39.5% (47 / 119) | NR | NR |
| **REFLECT I (2021)** | 43.0% (58 / 135)* | 58.1% (36 / 62) | NR | NR |
| **SENTINEL (2017)** | NR | NR | NR | NR |
| **MISTRAL-C (2016)** | NR | NR | NR** | NR** |
| **CLEAN-TAVI (2016)** | 100% (50 / 50)*** | 100% (50 / 50)*** | NR | NR |
| **DEFLECT III (2015)** | NR | NR | NR | NR |

**Abbreviations: CEP:** Cerebral Embolic Protection, **NR:** Not Reported

***** Predilatation rates for REFLECT I and REFLECT II represent the randomized CEP cohorts.

****** The MISTRAL-C trial reported a total of 5 cases of balloon dilatation (an overall trial rate of 7.6%), but did not provide the specific breakdown between the CEP and control groups.

******* Predilatation of the native aortic valve was mandated by the study protocol in the CLEAN-TAVI trial for all patients prior to valve implantation.


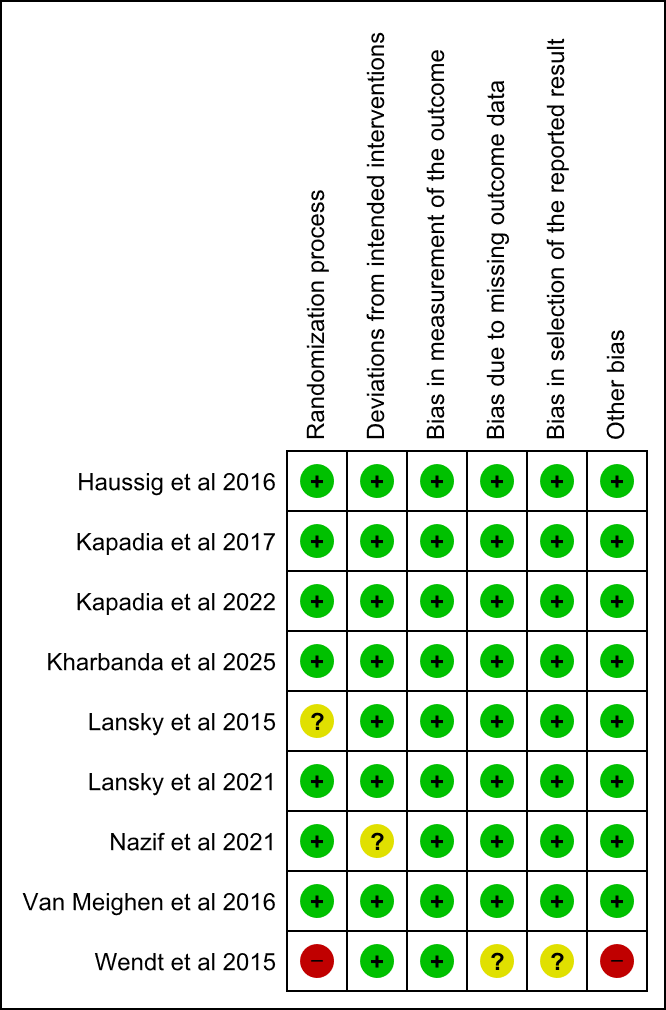


**Supplementary Figure 1:** Risk of bias assessment of randomized controlled trials.


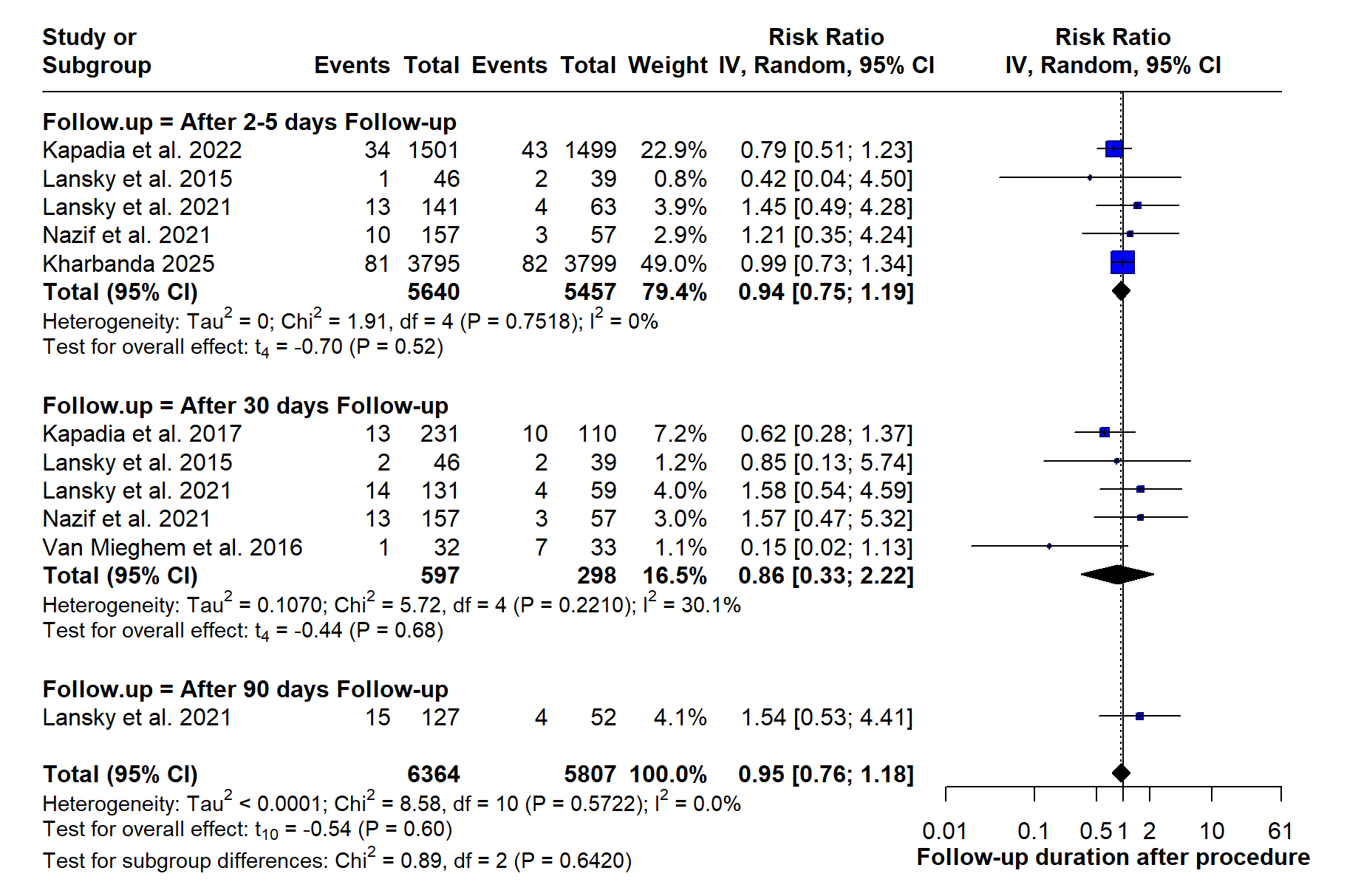


**Supplementary Figure 2:** Forest plot of stroke incidence according to the timing of occurrence during follow-up.


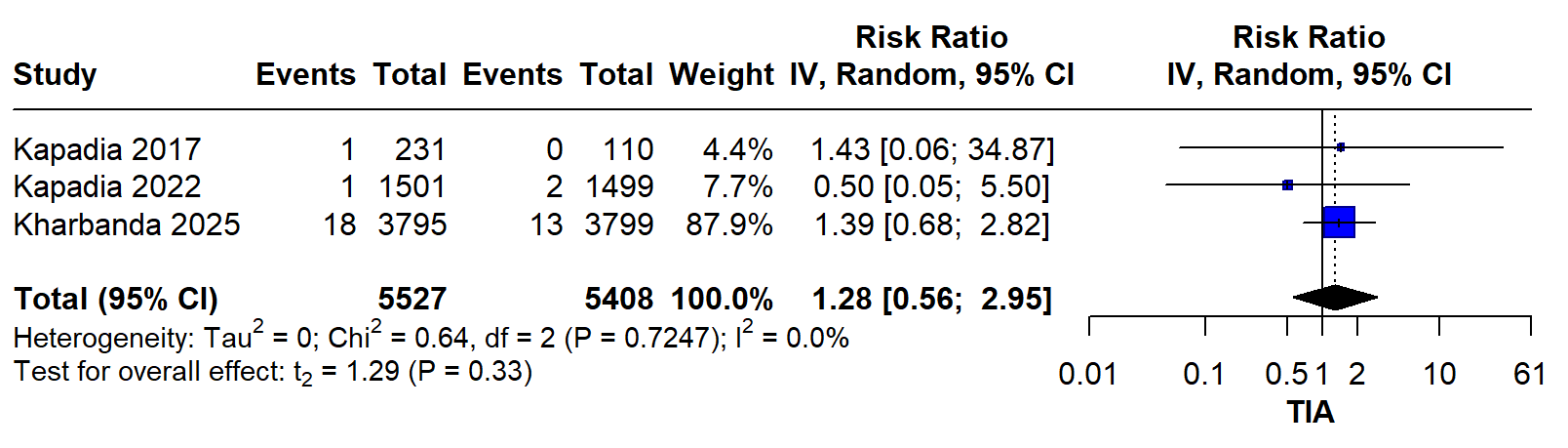


**Supplementary Figure 3:** Forest plot of incidence of transient ischemic attacks


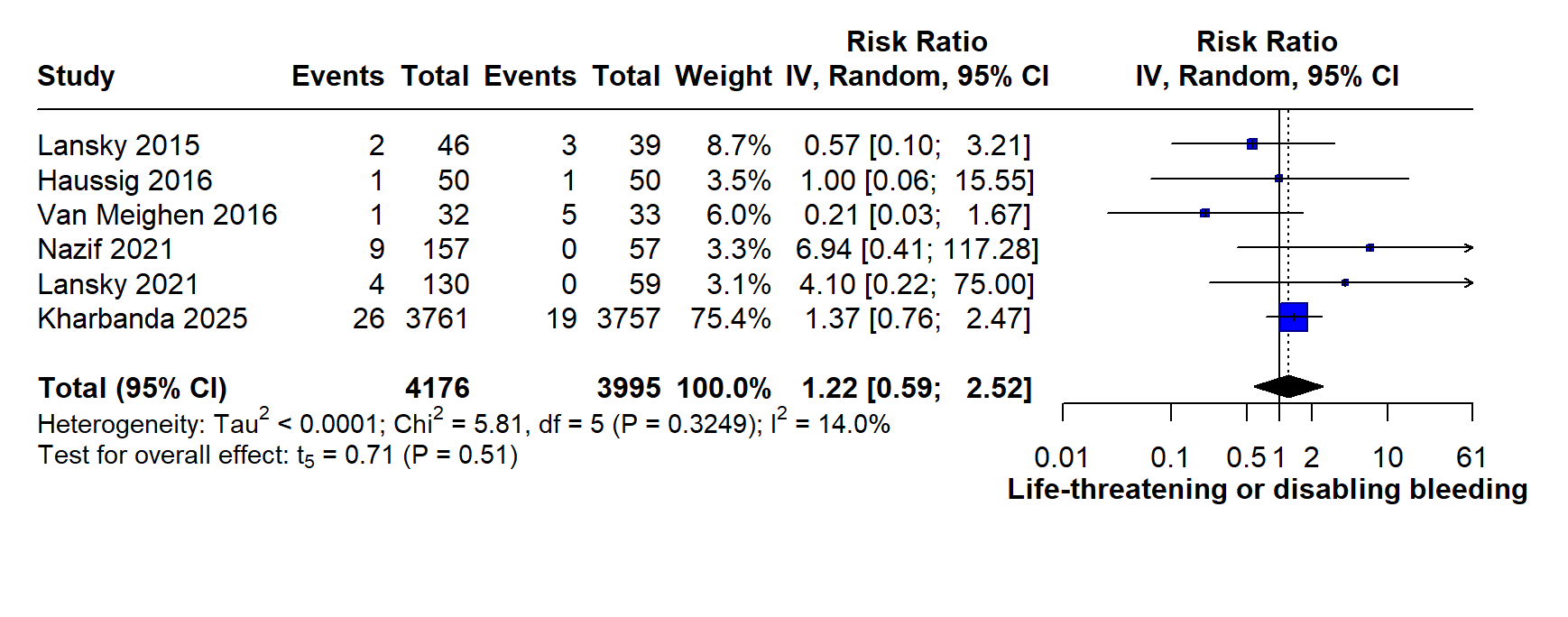


**Supplementary Figure 4:** Forest plot of incidence of life-threatening or disabling bleeding.


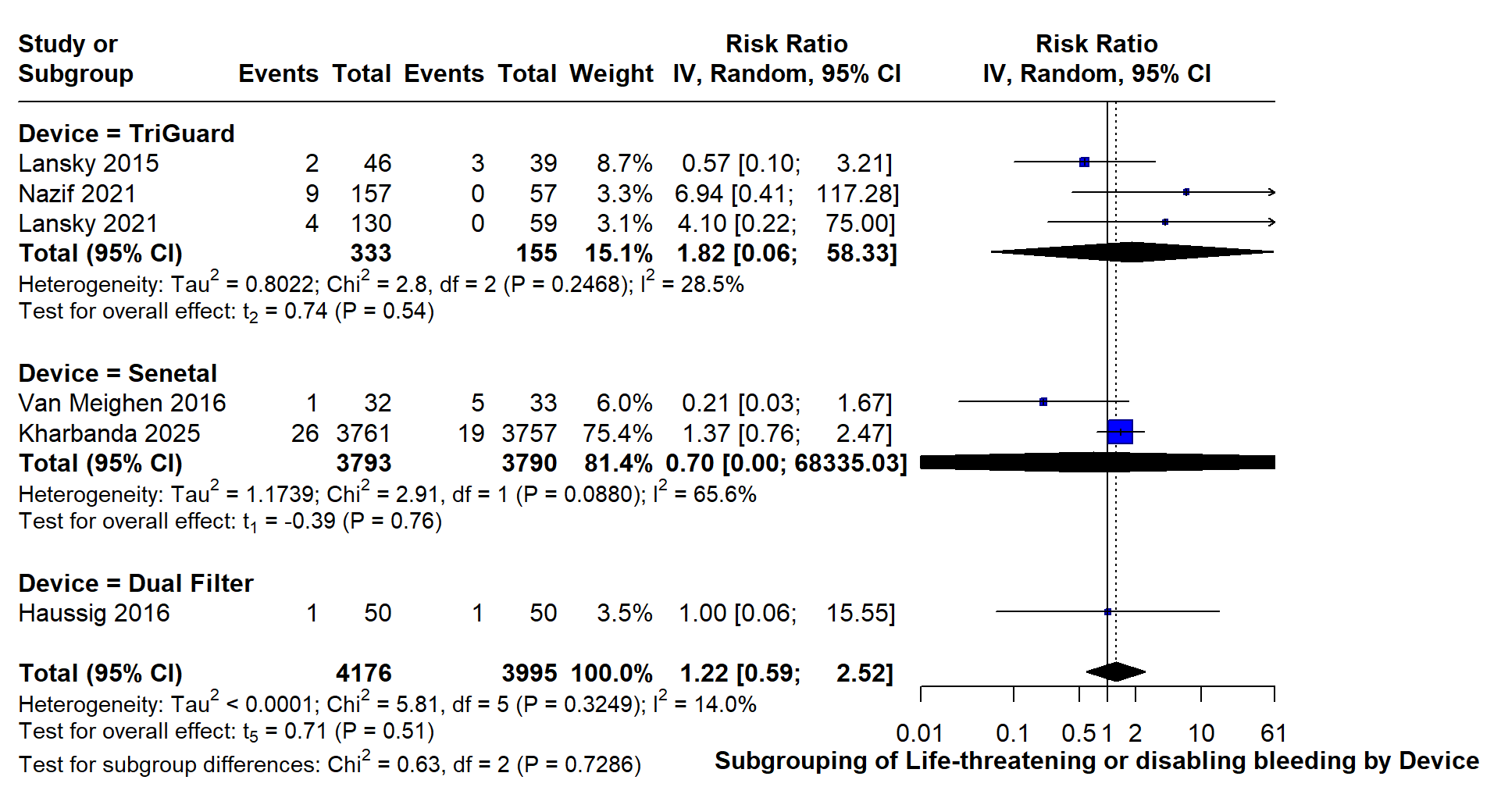


**Supplementary Figure 5:** Forest plot of subgroup analysis of the incidence of life-threatening or disabling bleeding by device.


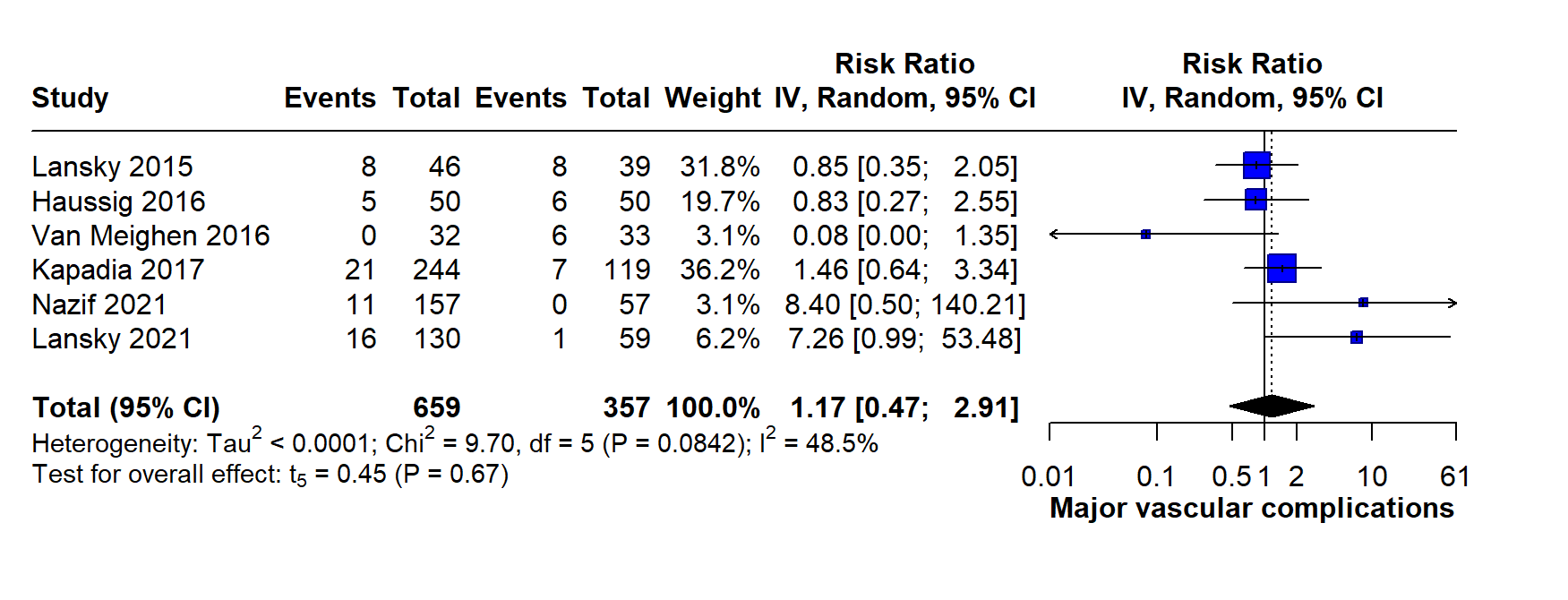


**Supplementary Figure 6:** Forest plot of the incidence of Major vascular complications.


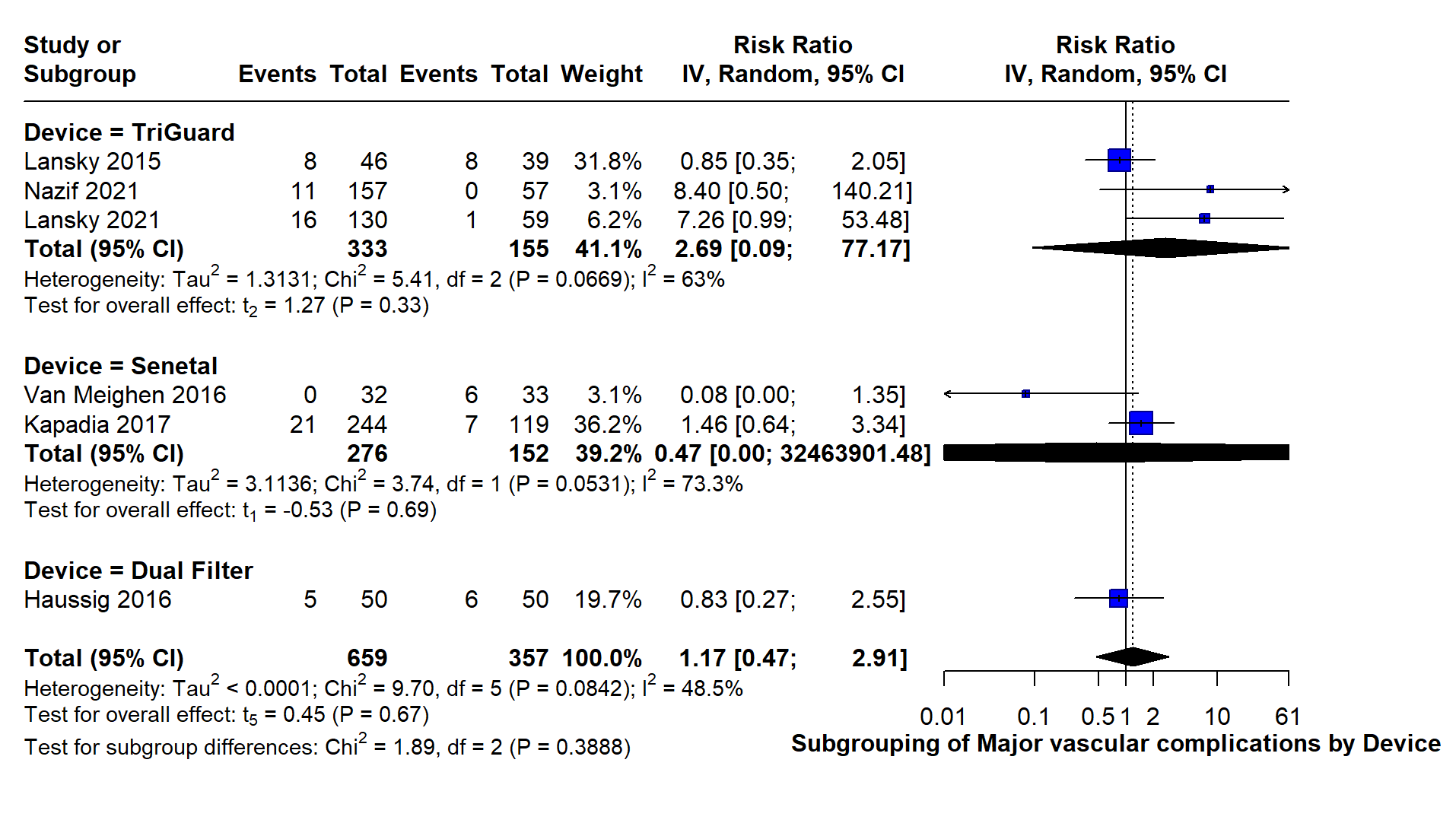


**Supplementary Figure 7:** Forest plot of subgroup analysis of the incidence of Major vascular complications by device.


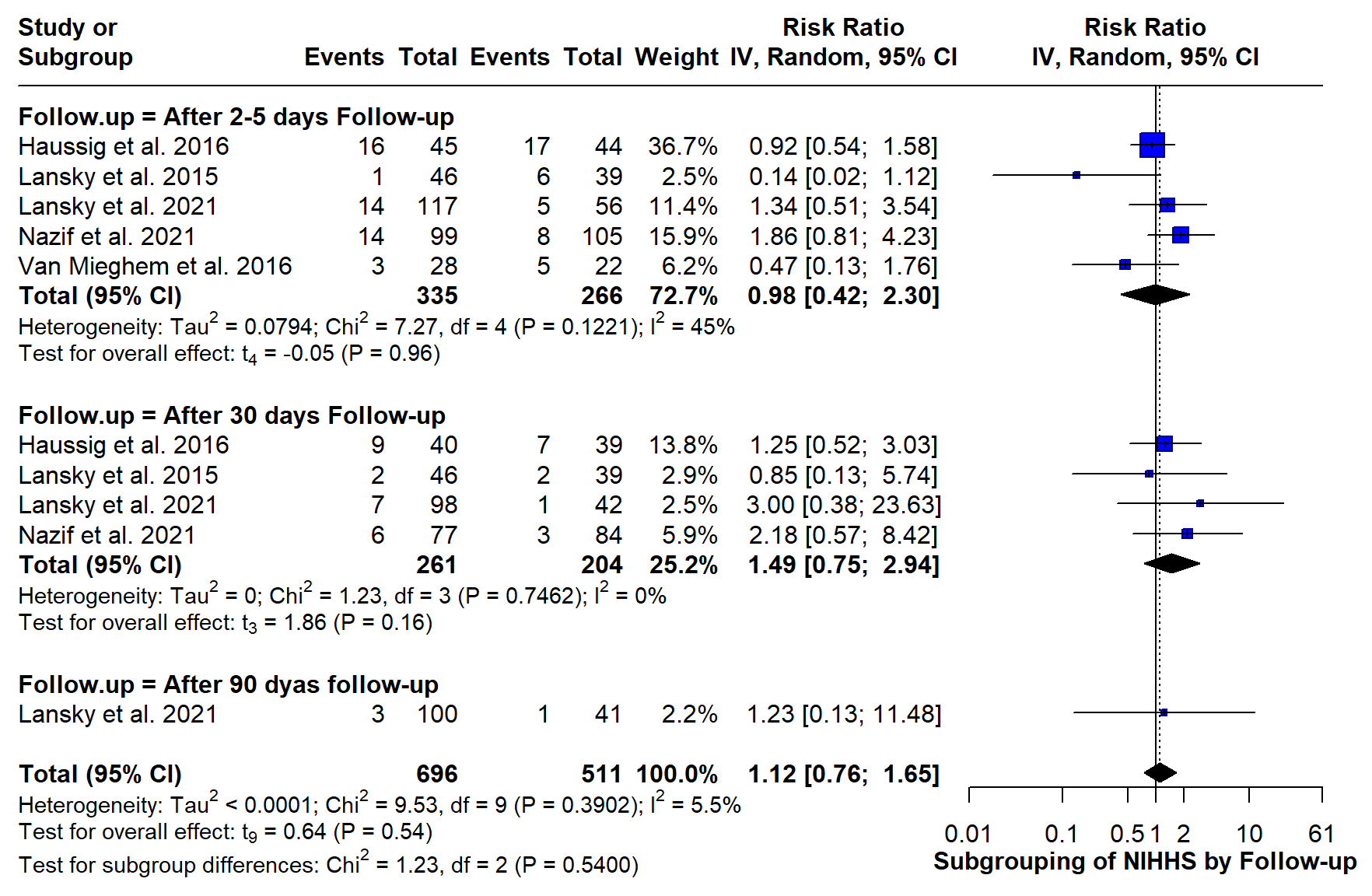


**Supplementary Figure 8:** Forest plot of National Institutes of Health Stroke Scale (NIHSS) Score Worsening.


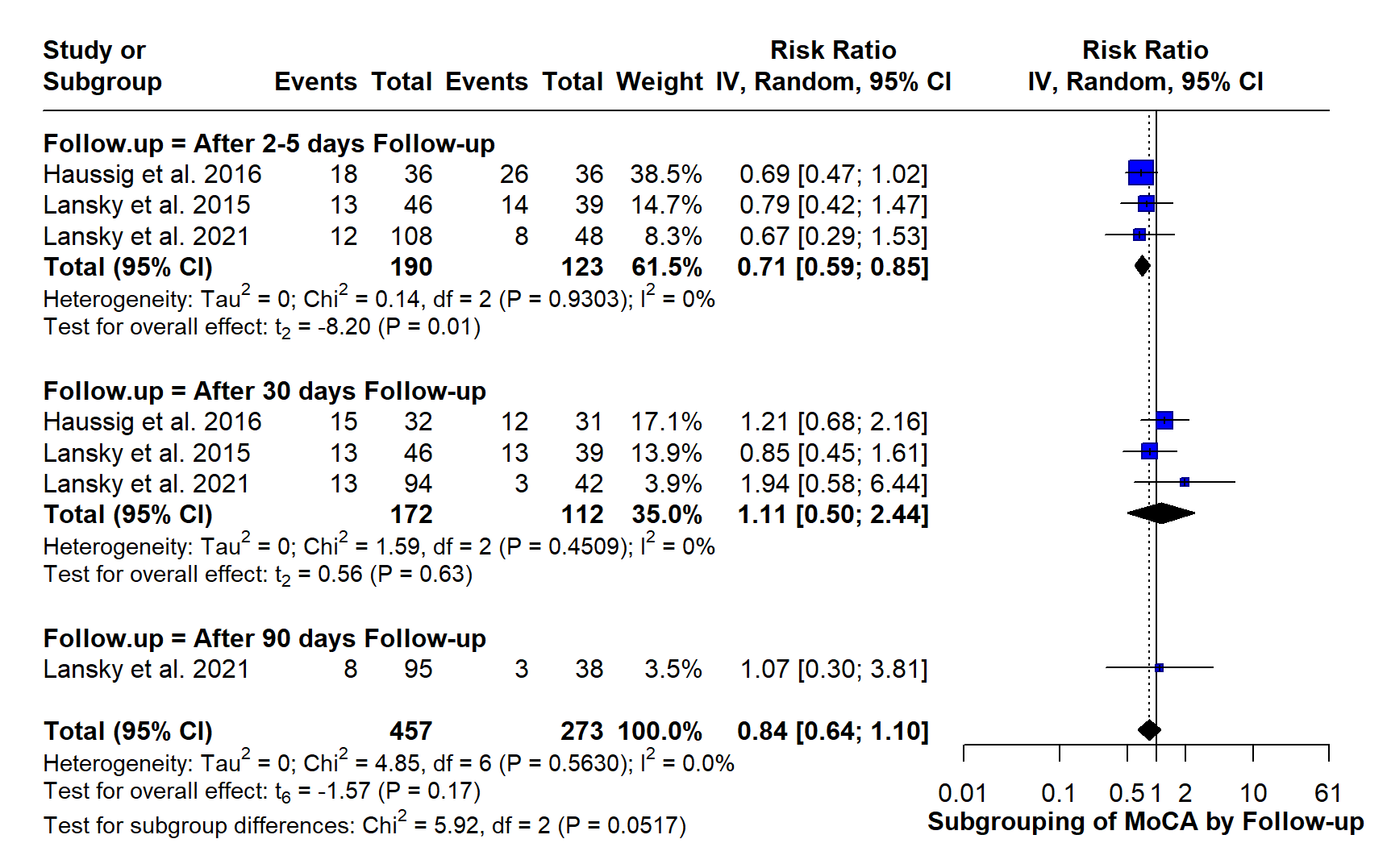


**Supplementary Figure 9:** Forest plot of Montreal Cognitive Assessment (MoCA) Score Worsening.


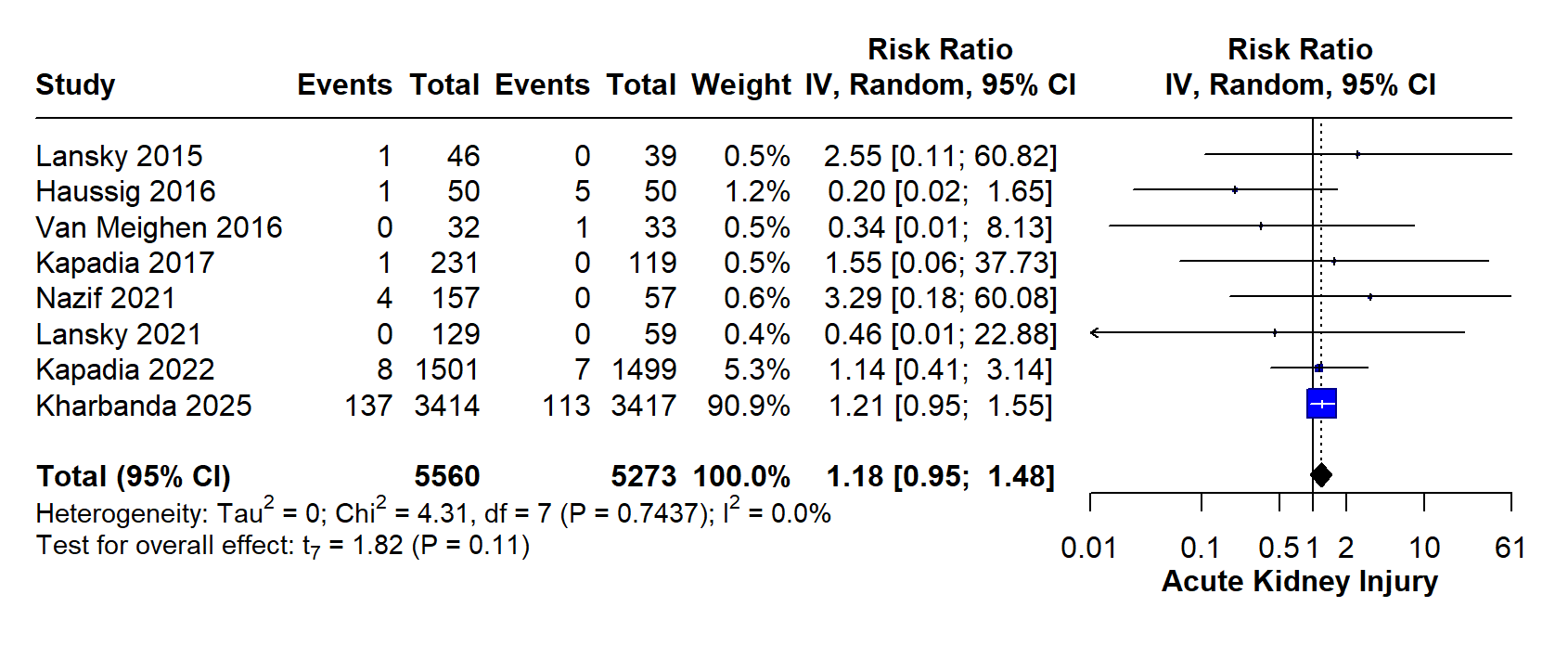


**Supplementary Figure 10:** Forest plot of the incidence of Acute Kidney Injury (AKI).


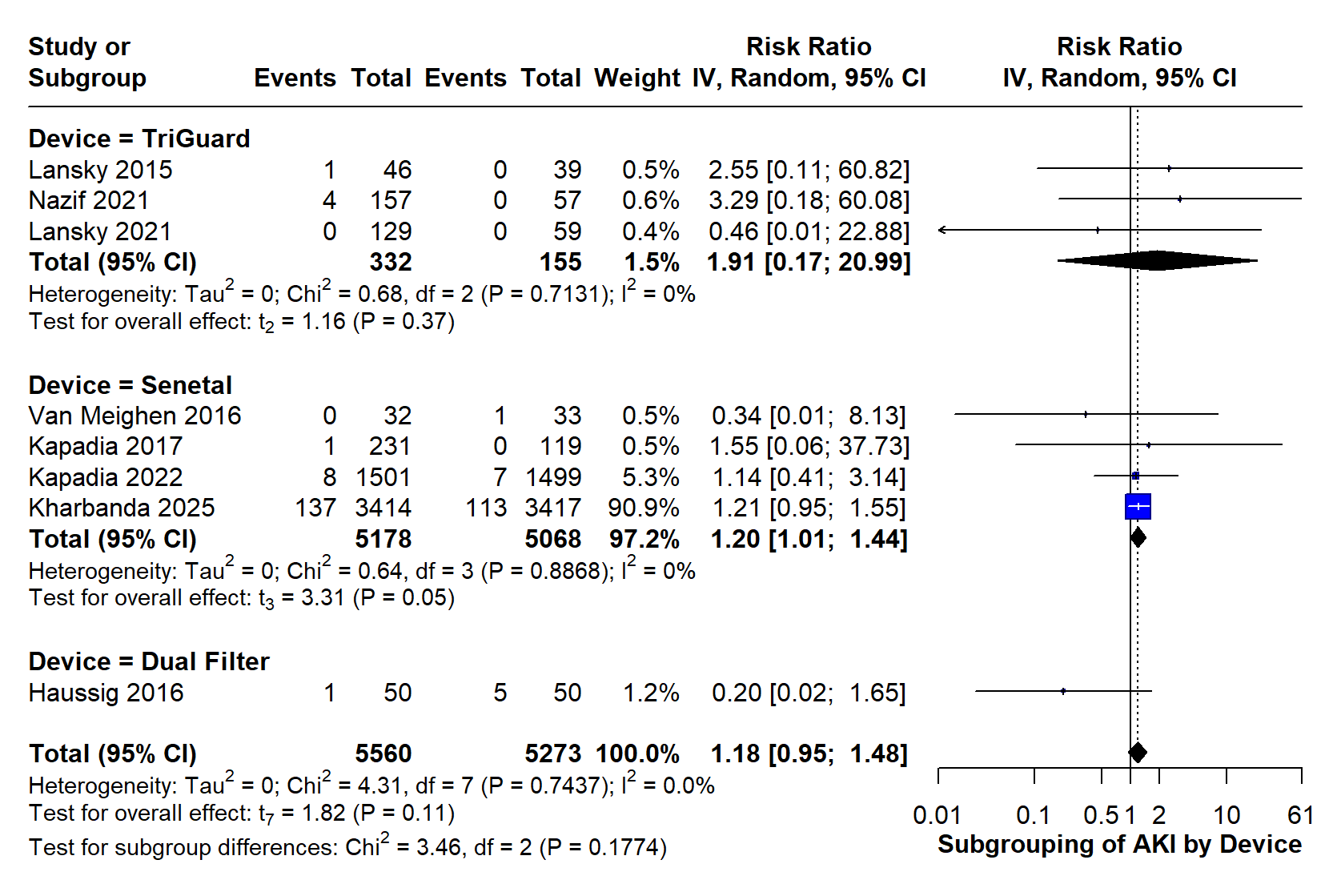


**Supplementary Figure 11:** Forest plot of subgroup analysis of the incidence of Acute Kidney Injury (AKI) by device.


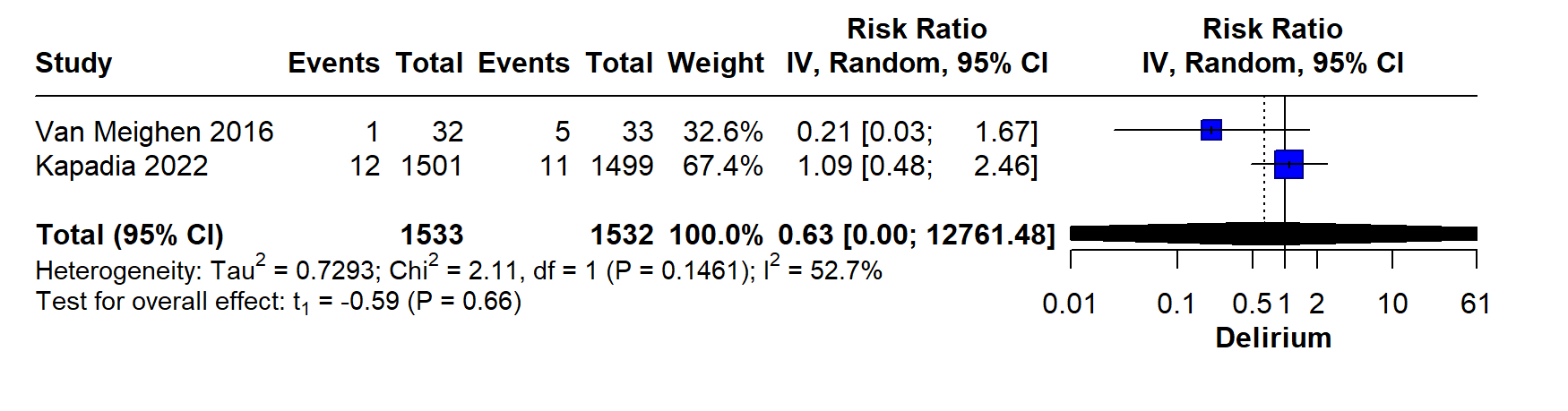


**Supplementary Figure 12:** Forest plot of the incidence of post-procedural delirium.


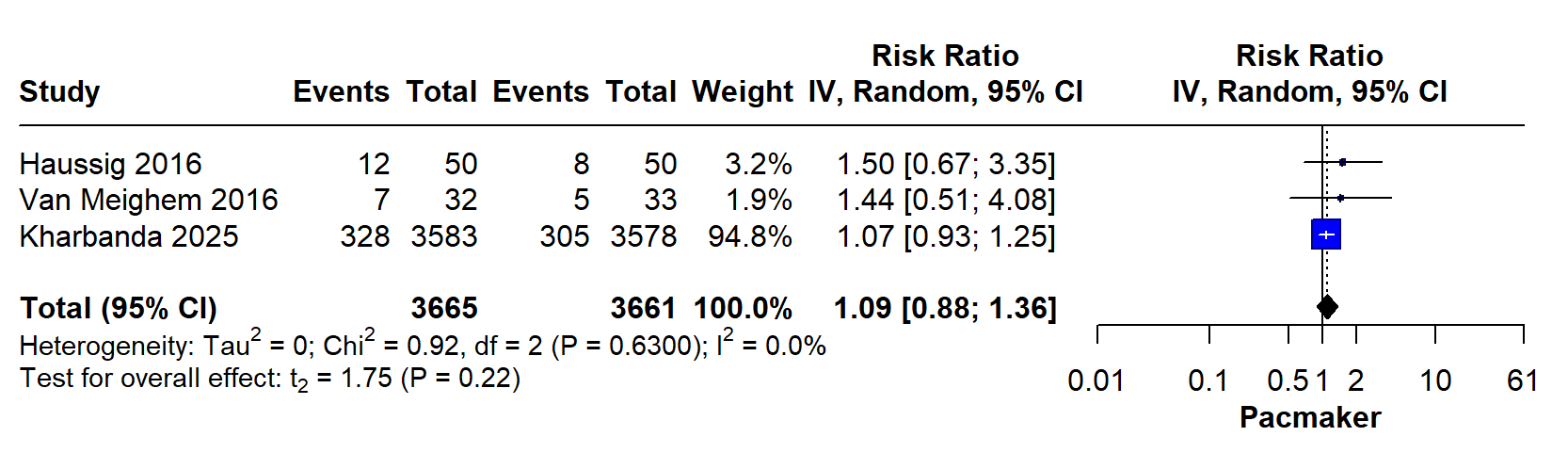


**Supplementary Figure 13:** Forest plot of the need for pacemaker implantation.

| **Section and Topic** | **Item #** | **Checklist item** | **Location where item is reported** |
| --- | --- | --- | --- |
| **TITLE** | | |  |
| Title | 1 | Identify the report as a systematic review. | 1 |
| **ABSTRACT** | | |  |
| Abstract | 2 | See the PRISMA 2020 for Abstracts checklist. | 2 |
| **INTRODUCTION** | | |  |
| Rationale | 3 | Describe the rationale for the review in the context of existing knowledge. | 3 |
| Objectives | 4 | Provide an explicit statement of the objective(s) or question(s) the review addresses. | 3 |
| **METHODS** | | |  |
| Eligibility criteria | 5 | Specify the inclusion and exclusion criteria for the review and how studies were grouped for the syntheses. | 3-4 |
| Information sources | 6 | Specify all databases, registers, websites, organisations, reference lists and other sources searched or consulted to identify studies. Specify the date when each source was last searched or consulted. | 3-4 |
| Search strategy | 7 | Present the full search strategies for all databases, registers and websites, including any filters and limits used. | 3-4 |
| Selection process | 8 | Specify the methods used to decide whether a study met the inclusion criteria of the review, including how many reviewers screened each record and each report retrieved, whether they worked independently, and if applicable, details of automation tools used in the process. | 3-4 |
| Data collection process | 9 | Specify the methods used to collect data from reports, including how many reviewers collected data from each report, whether they worked independently, any processes for obtaining or confirming data from study investigators, and if applicable, details of automation tools used in the process. | 3-4 |
| Data items | 10a | List and define all outcomes for which data were sought. Specify whether all results that were compatible with each outcome domain in each study were sought (e.g. for all measures, time points, analyses), and if not, the methods used to decide which results to collect. | 3-4 |
|  | 10b | List and define all other variables for which data were sought (e.g. participant and intervention characteristics, funding sources). Describe any assumptions made about any missing or unclear information. | 3-4 |
| Study risk of bias assessment | 11 | Specify the methods used to assess risk of bias in the included studies, including details of the tool(s) used, how many reviewers assessed each study and whether they worked independently, and if applicable, details of automation tools used in the process. | 3-4 |
| Effect measures | 12 | Specify for each outcome the effect measure(s) (e.g. risk ratio, mean difference) used in the synthesis or presentation of results. | 3-4 |
| Synthesis methods | 13a | Describe the processes used to decide which studies were eligible for each synthesis (e.g. tabulating the study intervention characteristics and comparing against the planned groups for each synthesis (item #5)). | 5 |
|  | 13b | Describe any methods required to prepare the data for presentation or synthesis, such as handling of missing summary statistics, or data conversions. | 5 |
|  | 13c | Describe any methods used to tabulate or visually display results of individual studies and syntheses. | 5 |
|  | 13d | Describe any methods used to synthesize results and provide a rationale for the choice(s). If meta-analysis was performed, describe the model(s), method(s) to identify the presence and extent of statistical heterogeneity, and software package(s) used. | 5 |
|  | 13e | Describe any methods used to explore possible causes of heterogeneity among study results (e.g. subgroup analysis, meta-regression). | 5 |
|  | 13f | Describe any sensitivity analyses conducted to assess robustness of the synthesized results. | 5 |
| Reporting bias assessment | 14 | Describe any methods used to assess risk of bias due to missing results in a synthesis (arising from reporting biases). | 5 |
| Certainty assessment | 15 | Describe any methods used to assess certainty (or confidence) in the body of evidence for an outcome. | 4-5 |
| **RESULTS** | | |  |
| Study selection | 16a | Describe the results of the search and selection process, from the number of records identified in the search to the number of studies included in the review, ideally using a flow diagram. | 5 |
|  | 16b | Cite studies that might appear to meet the inclusion criteria, but which were excluded, and explain why they were excluded. | 5 |
| Study characteristics | 17 | Cite each included study and present its characteristics. | 5 |
| Risk of bias in studies | 18 | Present assessments of risk of bias for each included study. | 5 |
| Results of individual studies | 19 | For all outcomes, present, for each study: (a) summary statistics for each group (where appropriate) and (b) an effect estimate and its precision (e.g. confidence/credible interval), ideally using structured tables or plots. | 6 |
| Results of syntheses | 20a | For each synthesis, briefly summarise the characteristics and risk of bias among contributing studies. | 6 |
|  | 20b | Present results of all statistical syntheses conducted. If meta-analysis was done, present for each the summary estimate and its precision (e.g. confidence/credible interval) and measures of statistical heterogeneity. If comparing groups, describe the direction of the effect. | 6 |
|  | 20c | Present results of all investigations of possible causes of heterogeneity among study results. | 6 |
|  | 20d | Present results of all sensitivity analyses conducted to assess the robustness of the synthesized results. | 6 |
| Reporting biases | 21 | Present assessments of risk of bias due to missing results (arising from reporting biases) for each synthesis assessed. | 5 |
| Certainty of evidence | 22 | Present assessments of certainty (or confidence) in the body of evidence for each outcome assessed. | 8 |
| **DISCUSSION** | | |  |
| Discussion | 23a | Provide a general interpretation of the results in the context of other evidence. | 9 |
|  | 23b | Discuss any limitations of the evidence included in the review. | 12 |
|  | 23c | Discuss any limitations of the review processes used. | 12 |
|  | 23d | Discuss implications of the results for practice, policy, and future research. | 11 |
| **OTHER INFORMATION** | | |  |
| Registration and protocol | 24a | Provide registration information for the review, including register name and registration number, or state that the review was not registered. | 3 |
|  | 24b | Indicate where the review protocol can be accessed, or state that a protocol was not prepared. | 3 |
|  | 24c | Describe and explain any amendments to information provided at registration or in the protocol. | NA |
| Support | 25 | Describe sources of financial or non-financial support for the review, and the role of the funders or sponsors in the review. | 13 |
| Competing interests | 26 | Declare any competing interests of review authors. | 13 |
| Availability of data, code and other materials | 27 | Report which of the following are publicly available and where they can be found: template data collection forms; data extracted from included studies; data used for all analyses; analytic code; any other materials used in the review. | 13 |
